# Awareness and practical evaluation of correct use of iron chelators; a study to track the ambiguities of thalassemia patients on their medications in Iran

**DOI:** 10.1186/s13104-024-06819-3

**Published:** 2024-06-13

**Authors:** Mina Saddat Mousavi, Ghader Mohammadnezhad, Farideh Yaghmaei, Azita Azarkeivan, Hadi Esmaily

**Affiliations:** 1 Department of Clinical Pharmacy, School of Pharmacy, Shahid Beheshti University of Medical Sciences, 3rd floor of the school of pharmacy, 2660th Valiasr Street, Niyayesh junction, Tehran, 1996835113 Iran; 2 Nursing and Midwifery School, Shahid Beheshti University of Medical Sciences, Tehran, Iran; 3 Blood Transfusion Research Center, High Institute for Research and Education in Transfusion Medicine, Thalassemia Clinic, Tehran, Iran

**Keywords:** Thalassemia, Iron chelators, Drug utilization, Transfusion, Patient awareness

## Abstract

**Purpose:**

This study aimed to evaluate the knowledge, attitude, and practice toward iron chelating agents (ICAs) in Iranian thalassemia major patients.

**Methods:**

A total of 101 patients with thalassemia major were involved in this cross-sectional survey. A deep medication review was done, and participants’ knowledge, attitude, and practice were evaluated by a validated instrument based on a 20-scoring system.

**Results:**

Statistical analyses showed 52 patients (51.5%) had a poor knowledge level (scores < 10) about their medications, 37 (36.6%) had a moderate level (scores 10–15), and 12 (11.9%) had a satisfactory level (scores > 15). Seventy-seven (76.2%) patients have positive beliefs regarding the dependence of their current health status on taking iron chelators, and 63 (62.4%) believed that they would become very ill without taking medication. The results also showed that the mean practice score in patients who received deferoxamine was 5.81 ± 3.50; in the patients who received deferiprone and those who received deferasirox, the mean scores were 7.36 ± 5.15 and 14.94 ± 4.14. Also, the knowledge and practice level had a direct linear correlation based on the regression analyses (*P* < 0.001).

**Conclusion:**

In conclusion, results of the present research suggests that the patients’ knowledge about the administration, adverse events, and necessity of ICAs was not satisfactory. Improving the knowledge of thalassemia patients toward their medicines through educational interventions is highly recommended to improve their practice level.

**Supplementary Information:**

The online version contains supplementary material available at 10.1186/s13104-024-06819-3.

## Background

Thalassemia is one of the most prevalent diseases in the group of inherited hemoglobinopathies. It is characterized by disrupting the standard ratio of alpha/beta globin production due to the mutation in the globin synthesis genes. This mutation leads to various phenotypes ranging from clinically asymptomatic conditions to severe life-threatening anemia [1]. Epidemiological studies have demonstrated that annually, almost 70,000 infants are born with β-thalassemia worldwide [2]. In Iran, about 25,000 people suffer from thalassemia major [3]. In the past decade, the survival rate in these patients increased significantly due to better treatment and supportive care [4]. Blood transfusion and iron chelating agents (ICAs) are the cornerstones of thalassemia management [5–7]. ICAs like Deferoxamine (DFX), Deferiprone (DFP), and Deferasirox (DFS) are lifelong interventions in these patients [8]. Adherence to medications will be crucial in such a chronic condition. Evaluating the knowledge, attitude, and practice (KAP) of thalassemia patients about their medications provides valuable information for policymakers to predict other aspects of healthcare principles other than providing their medications to improve the health status of these patients [9, 10]. Also, the abovementioned factors can alter their adherence to the treatment. Because ICAs are self-administered, a poor level of practice can lead to the failure of medications and increase unnecessary health costs, morbidity, and mortality [11]. Hence, it is required to evaluate the involved factors to make appropriate strategies for increasing knowledge and improving attitude and practice [12].

## Methods

### Survey overview

This cross-sectional study was conducted between April 2022 and January 2023 in Tehran, Iran. These patients were under treatment with ICAs and could read and speak Persian. Patients signed the informed consent form and the Shahid Beheshti University of Medical Sciences ethics committee (IR.SBMU.PHARMACY.REC.1398.044). The Iranian Association of Thalassemia approved the protocol of the study.

### Sample size and sampling method

Shahid Kazemi Pharmacotherapy Clinic had a private registry of all patients with Thalassemia Major. We used the simple random sampling method. After informing the patient and describing all aspects of the survey, based on the Helsinki Declaration we requested them to participate in the survey after signing the informed consent form. In the mentioned registry, there was complete information on 143 patients with thalassemia major, and based on Morgan et al.‘s sample size estimation table, 103 participants could have been included in the study.

### Questionnaires and data extraction

We used a valid and reliable questionnaire to assess the knowledge and practice of thalassemia patients toward ICAs. The beliefs about medicines questionnaire (BMQ-18) was used in the current study for evaluating attitudes. This questionnaire is well-known because it evaluates the main aspects of beliefs about medications, including harm, overuse, necessity, and concern. The questionnaire consists of two parts; general and specific. The questions of the general part explore two sections regarding over-prescribing by physicians (five questions, scores ranging from 5 to 25) and the harmfulness of medicines (four questions, scores ranging from 5 to 20). Also, the questions of the specific part include two sub-scales, the necessity of medicines (five questions, score ranges from 5 to 25), and concerns of adverse drug reactions (ADR) (four questions, score ranges from 5 to 20). Based on the Likert scale, high scores in the “necessity” section represent the more robust perception of taking medicines to maintain health. Higher scores in the “concerns” section indicate more concern regarding the adverse effects of medicines. The high scores in the “harm” section showed a more negative perception of the medicine. Finally, higher scores in the “overuse” section represent physicians’ more negative attitudes toward medicine prescription trends. Also, because monitoring ADR is a crucial matter of concern in this study, we added another question to BMQ-18 to the “concern” section that assesses the tolerance to ADR. Each item was scored on a five-point Likert scale (1- strongly disagree, 2- disagree, 3- no comments, 4- agree, and 5- strongly agree) [13]. Scores obtained for the individual items within the scale were summed up to generate total scores. Higher scores indicated stronger beliefs. To define the limits of measurement to evaluate the level of knowledge, the expert panel defined the cut-off of the level of knowledge based on the data obtained from the survey. In this stratification, up to 6 points were considered as poor knowledge, 6 to 14 as moderate level, and more than 14 as acceptable knowledge. Also, demographic variables such as age, sex, marital status, education level, blood group, concurrent co-morbidities, family history, blood transfusion intervals, food/drug allergy, current health status, and medication records were collected [14]. The questionnaire details were shown in the supplementary file 1 and 2.

### Transcription

The recorded conversation verbatim was transcribed in the first step. Then conversations were in Persian, so it was translated into English by employing a forward and backward translation technique to maintain concepts and validity in the publication of results.

### Statistical analyses

Data analysis was performed using SPSS software (v.25.0; SPSS Inc., Chicago, IL, USA). The Kolmogorov-Smirnov test checked the normal distribution and parametric data. The Chi-Square test was used to examine the relationship between non-parametric variables. Qualitative data are expressed as mean ± SD and to compare the groups, Analysis of Variance (ANOVA) was used. In addition, the linear regression model was used to evaluate the association between the knowledge level and the blood transfusion intervals. Also, the association between knowledge and practice levels in patients with thalassemia. The *P* < 0.05 was considered significant in comparing means and correlations.

## Results

This study was carried out to assess the KAP level in Iranian thalassemia patients. Participants were 101 thalassemia patients, of whom 43 (42.6%) were men and 58 (57.4%) were women. The mean age of participants was 29.65 ± 6.44. Among the patients, 43 (42.6%) received DFX, 32 (31.7%) received DFP, and 26 (25.7%) received DFS. The majority of patients (72.25%) were single. The sociodemographic characteristics of the participants are presented in Table 1. Regarding educational status, 54 (53.46%) had an academic degree, 38 patients (37.62%) had a diploma, and 9 (8.91%) had a lower educational degree. In 43 (42.57%) patients, the disease was diagnosed during childhood.

**Table 1:** Demographic characteristics of participants

Demographic Parameter		Results
Age (years old)		29.65 ± 6.44
Sex	Female	58 (57.40%)
	Male	43 (42.60%)
Education	Under Diploma	9 (8.91%)
	Diploma	38 (37.62%)
	University Degree	54 (53.46%)
Marital Status	Single	76 (75.25%)
	Married	25 (24.75%)
Co-Morbidities	No co-morbidity	54 (53.46%)
	Diabetes	16 (15.84%)
	Hypothyroidism	9 (8.91%)
	Osteoporosis	6 (5.94%)
	Heart failure	4 (3.96%)
	Depression	2 (1.98%)
	Lupus	2 (1.98%)
	Chronic viral hepatitis	2 (1.98%)
	Hypogonadism	2 (1.98%)
	Others (less than 1%)	4 (3.96%)
Family History of Thalassemia	Yes	78 (77.23%)
	No	23 (22.77%)

**Fig. 1:**
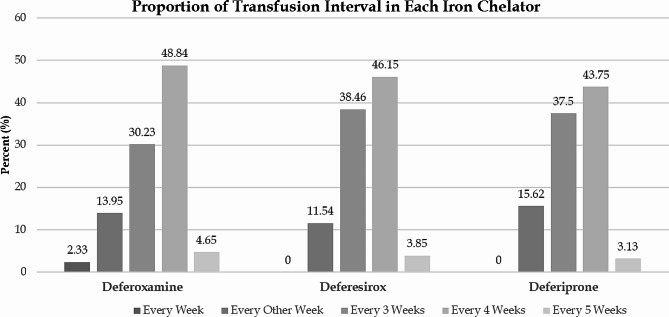
The blood transfusion interval in each group of ICAs. There was no statistically significant difference between ICAs in the point of blood transfusion interval (*P* = 0.996). [ICAs: iron chelating agents, DFX: deferoxamine, DFP: deferiprone, DFS: deferasirox]

With a 15.84% incidence, diabetes was the most prevalent co-morbidity among participants. The results of the blood transfusion interval in each ICA are presented in Fig. 1. There was no statistically significant difference between ICAs in the point of blood transfusion interval (*P* = 0.996).

Fifty-two patients (51.5%) had a poor knowledge level about thalassemia medication, 37 (36.6%) had moderate knowledge, and 12 (11.9%) had an acceptable level of knowledge. There was no statistically significant difference between the knowledge of patients who received DFX and DFP (*P* = 0.989). However, there was a statistically significant difference between the knowledge of patients who received DFS and DFP (*P* < 0.001).

Seventy-seven (76.2%) patients believed their current health depends on their medication, and 63 (62.4%) believed they would become very ill without medications. Concerns about ICAs were high; 75 (74.3%) worried about the long-term effects of their medicines, and 35 (34.7%) believed thalassemia medications could disrupt their life. Forty-three (42.6%) believed ICAs were a “mystery” to them. Thirty-nine (38.6%) patients believed that most medicines are addictive. Moreover, 45 (44.6%) patients reported that medicines incur more harm than benefits. Seventy-five (74.2%) believed that doctors use too many medicines, and 67 (66.3%) patients believed that if they had more time with doctors, they would prescribe fewer medicines. The results of attitude with more details are summarized in Table 2.

**Table 2:** Attitude toward iron chelating agents in Iranian thalassemia patients

BMQ subscales	Mean ± SD			Patients scoring above midscale (%)
	Deferoxamine	Deferiprone	Deferasirox	
**Harm**				
All medicines are poisons	3.12 ± 1.34	2.96 ± 1.01	3.23 ± 1.17	18.31
Medicines do more harm than good	2.83 ± 1.33	2.65 ± 1.39	2.88 ± 0.94	
Most medicines are addictive	2.93 ± 1.26	2.81 ± 1.38	3.11 ± 0.99	
People who take medicines should stop their treatment for a while now and again	3.41 ± 1.16	3.12 ± 1.22	3.15 ± 1.17	
**Overuse**				
Doctors use too many medicines	2.09 ± 0.88	2.34 ± 1.03	2.38 ± 0.94	37.12
Natural remedies are safer than medicines	2.06 ± 1.28	2.50 ± 1.35	2.38 ± 1.33	
Doctors place too much trust in medicines	1.90 ± 0.58	1.87 ± 0.49	2.00 ± 1.19	
If doctors had more time with patients, they would prescribe fewer medicines	2.67 ± 0.92	2.15 ± 0.51	2.34 ± 0.69	
**Necessity**				
My health, at present, depends on my thalassemia medicines.	2.11 ± 1.36	2.15 ± 1.39	1.76 ± 1.05	34.56
My life would be impossible without my thalassemia medicines.	2.77 ± 1.10	2.59 ± 1.13	2.23 ± 1.07	
Without my thalassemia medicines, I would be very ill.	2.51 ± 1.32	2.53 ± 1.15	2.38 ± 1.16	
My health in the future will depend on my thalassemia medicines	2.51 ± 1.49	2.25 ± 1.47	2.07 ± 1.03	
My thalassemia medicines protect me from becoming worse.	1.84 ± 1.29	2.12 ± 1.65	1.73 ± 1.19	
**Concern**				
Having to take thalassemia medicines worries me.	2.35 ± 0.79	2.75 ± 0.94	2.88 ± 1.03	27.23
I sometimes worry about the long-term effects of my thalassemia medicines.	2.22 ± 1.02	2.50 ± 1.09	1.81 ± 0.48	
My thalassemia medicines are a mystery to me.	2.96 ± 1.10	2.84 ± 0.94	2.80 ± 1.22	
My thalassemia medicines disrupt my life.	2.71 ± 1.18	3.03 ± 1.27	2.88 ± 1.03	
I sometimes worry about becoming too dependent on my thalassemia medicines.	2.13 ± 0.75	2.25 ± 0.83	2.77 ± 0.79	
Taking these medications gives me worry about adverse drug reactions.	2.09 ± 0.92	2.21 ± 0.94	2.76 ± 1.15	

The subgroup analysis was performed to explore the differences in attitude aspects between ICAs, which showed no significant differences. These results also are summarized in Table 3.

**Table 3:** Attitude toward ICAs in thalassemia patients based on different aspects

BMQ subscales (Out of 20)	Mean ± SD			*P*
	Deferoxamine	Deferiprone	Deferasirox	
Necessity	9.77 ± 3.39	10.12 ± 3.20	8.80 ± 2.64	0.220
Concern	9.62 ± 2.22	10.54 ± 2.45	10.79 ± 3.20	0.134
Overuse	8.32 ± 2.28	8.94 ± 2.45	8.61 ± 2.25	0.538
Harm	11.81 ± 3.24	11.38 ± 3.54	12.48 ± 2.79	0.406

The results also showed that the mean practice score (out of 20) in patients who received DFX was 5.81 ± 3.50. In the patients who received DFP and those who received DFS, the mean score was 7.36 ± 5.15 and 14.94 ± 4.14, respectively. The mean practice score significantly differs between these three groups (*P* < 0.001). Also, analysis of variance in Table 4 on education levels showed that people with higher education have significantly higher knowledge and practice about their ICAs (*P* < 0.001).

**Table 4:** Knowledge and Practice Levels among the Population with Different Levels of Education

		Mean ± SD	95%CI for Mean
Knowledge	Under Diploma	5.00 ± 1.4	4.44–5.56
	Diploma	8.78 ± 0.9	8.39–9.17
	University Degree	15.85 ± 2.8	15.05–16.64
Practice	Under Diploma	5.25 ± 2.8	4.94–6.56
	Diploma	9.43 ± 2.9	8.03–11.90
	University Degree	12.56 ± 6.8	10.25-18.00

**Fig. 2:**
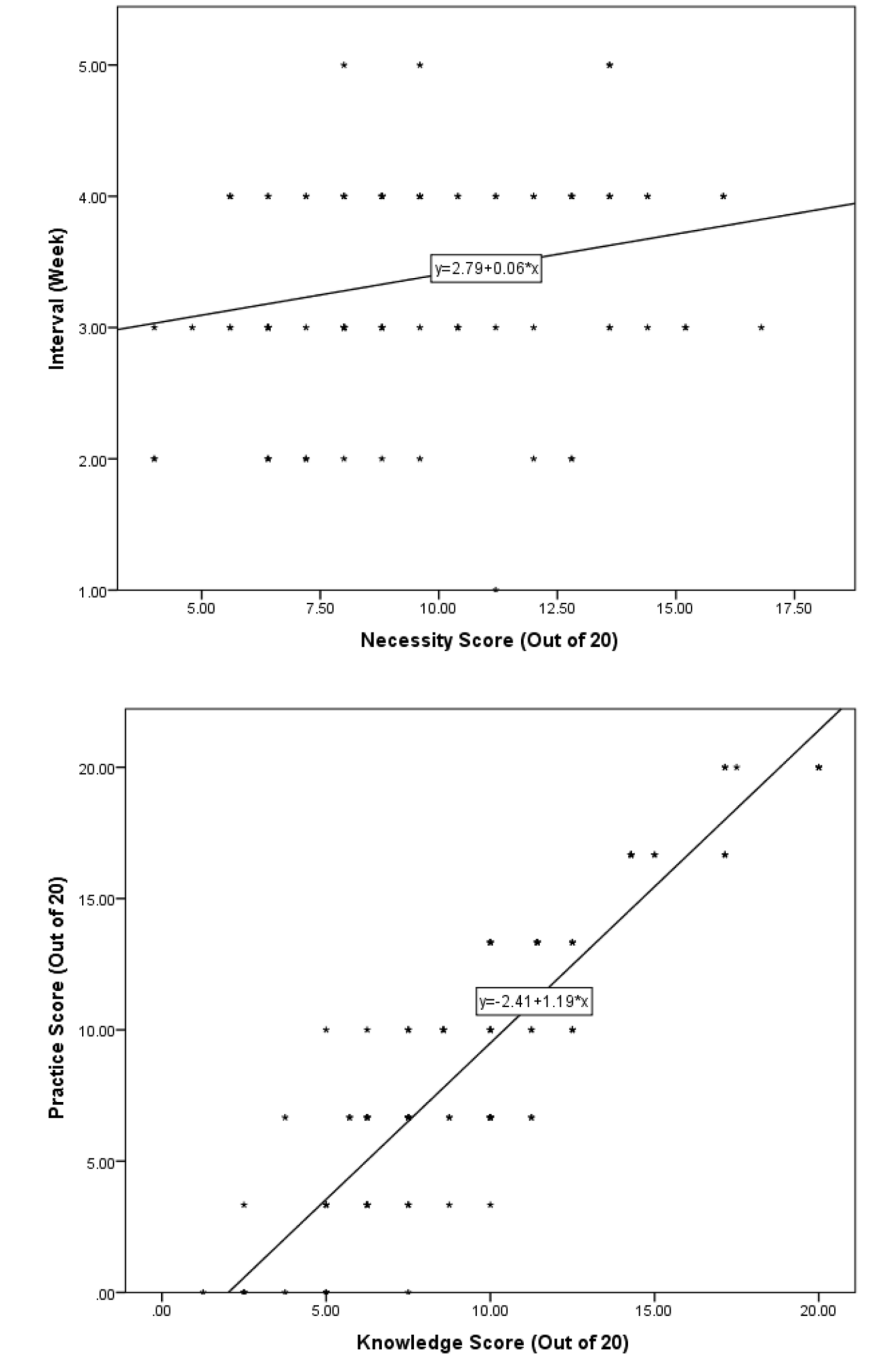
The correlations between the necessity of blood transfusion versus interval (**A**), and knowledge score versus practice score (**B**). The linear regression results showed a direct correlation between necessity and blood transfusion interval (*P* = 0.009). Also, a significant direct correlation was detected between knowledge level and practice score (*P* < 0.001)

The linear regression results showed a significant direct relation between necessity and blood transfusion interval (*P* = 0.009). Also, a significant direct relation was detected between knowledge level and practice score (*P* < 0.001). These relations are presented in Fig. 2.

## Discussion

Thalassemia is a chronic congenital disorder that causes physical and psychological co-morbidities [15]. This disorder not only affects the patient but also has a negative impact on the patient’s family and a considerable burden on the health budget of countries, especially those on the thalassemia belt [16–18]. Based on the results of this study, most patients’ knowledge was not acceptable regarding their medications. However, patients who received DFS had a higher level of knowledge compared with other patients. In Iran, the manufacturer of DFS holds educational courses to improve patient information. Hence, this finding may be due to this intervention, and it can be concluded that such educational courses are beneficial tools for enhancing knowledge levels. Also, the attitude of patients was not satisfactory toward their medications. Patients who received DFS had a strong perception of their medications. This also may be due to their educational courses.

Although knowledge improvement affects the attitude level, this study showed that improving patients’ knowledge could increase their concerns regarding their medicines. However, the comparison between DFS and other groups was not significantly different. In the practice area, patients who received DFS had a high practice score, suggesting that their knowledge and attitude shaped their practice. Also, the interval of blood transfusion is not significantly different between ICA groups. However, further analysis by linear regression illustrated a significant direct relationship between the perception of necessity score and the blood transfusion interval of patients. This means that patients with a high perception of necessity regarding their medicines may need less blood transfusion. The patients with a high level of knowledge had better practice regarding their medicines. The abovementioned relations can be used as an instrument for policymakers to plan effective strategies for KAP improvement among these patients. Any strategies that increase the blood transfusion interval will be highly cost-effective for health systems other than decreasing the complications of thalassemia mismanagement. A study conducted by Kourorian et al. in Tehran aimed to evaluate the KAP of thalassemia patients regarding their disease [19]. They deeply interviewed 190 patients with thalassemia and found that patients with a low knowledge level about thalassemia had an irregular and improper treatment course. Our findings also confirmed their results in this regard. The current study had a direct relationship between knowledge and practice. Abu Samra showed a significant relationship between the patient’s knowledge of the treatment with ICAs and their quality of life [18]. As mentioned earlier, thalassemia treatment has a significant burden in the thalassemia belt, and procurement of various blood types requires high costs [20]. Improving these patients’ KAP could lead to better insights for self-monitoring, which decreases their disease burden on the health system. Considering the impact of drug counseling on the health-related quality of life, encouraging patients’ positive attitude towards their drugs, and cost-savings related to the management of ADRs, it can be suggested for future studies that economic assessments and budget impact analyses evaluate the effectiveness of good pharmacy and clinical practice interventions and evaluation the rational use of ICAs [21–23]. In this survey, it was tried to control predictable confounding factors so that they cannot affect the results of the study, also if these factors are unpredictable, they have a homogenous distribution between the groups. In addition to the strengths, this study also has limitations that, like confounding factors, were tried to have the least impact on the results and data analysis. Among the limitations of this study, it was a single center, the impossibility of monitoring all thalassemia major patients in Iran, and comprehensive monitoring of their drug therapy. Also, long-term monitoring of patients was not possible.

## Conclusions

In conclusion, the present study suggests that the patients’ KAP toward ICAs was unsatisfactory. Knowledge and necessity perception are the most influencing parameters to improve the practice and blood transfusion interval. Hence, improving the knowledge and perception of thalassemia patients toward their medicines through educational interventions is highly recommended as a cost-effective strategy.

### Electronic supplementary material

Below is the link to the electronic supplementary material.

Supplementary Material 1

Supplementary Material 2

## Data Availability

No datasets were generated or analysed during the current study.

## References

[1] Galanello R, Origa R (2010). Beta-thalassemia. Orphanet J Rare Dis.

[2] Tritipsombut J, Phylipsen M, Viprakasit V, Chalaow N, Sanchaisuriya K, Giordano PC (2012). A single-tube multiplex gap-polymerase chain reaction for the detection of eight β-globin gene cluster deletions common in Southeast Asia. Hemoglobin.

[3] Khodaei GH, Farbod N, Zarif B, Nateghi S, Saeidi M (2013). Frequency of thalassemia in Iran and Khorasan Razavi. Int J Pediatr.

[4] Pearson H, Giardina P, Cohen A, Kazazian H, editors. The changing profile of homozygous beta-thalassemia. pediatric research; 1995: Williams & Wilkins 351 West Camden ST, BALTIMORE, MD 21201 – 2436.

[5] Brittenham GM, Griffith PM, Nienhuis AW, McLaren CE, Young NS, Tucker EE (1994). Efficacy of deferoxamine in preventing complications of iron overload in patients with Thalassemia major. N Engl J Med.

[6] Telfer P, Coen PG, Christou S, Hadjigavriel M, Kolnakou A, Pangalou E (2006). Survival of medically treated Thalassemia patients in Cyprus. Trends and risk factors over the period 1980–2004. Haematologica.

[7] Olivieri NF, Brittenham GM (1997). Iron-chelating therapy and the treatment of Thalassemia. Blood.

[8] Neufeld EJ (2006). Oral chelators deferasirox and deferiprone for transfusional iron overload in Thalassemia major: new data, new questions. Blood.

[9] Mohammadnezhad G, Sattarpour M, Azadi Kakavand M (2023). Community pharmacists’ practice regarding vitamin D products: a simulated client method. J Patient Saf Qual Improv.

[10] Cao A, Rosatelli MC, Monni G, Galanello R (2002). Screening for thalassemia: a model of success. Obstet Gynecol Clin.

[11] Ansari S, Baghersalimi A, Azarkeivan A, Nojomi M, Rad AH (2014). Quality of life in patients with Thalassemia major. Iran J Pediatr Hematol Oncol.

[12] Kosarian M, Vallee N, Mahdyanee A (2000). Do the desferal thalassemic patients have zinc deficiency receiver?. J Mazandaran Univ Med Sci.

[13] Horne R, Weinman J, Hankins M (1999). The beliefs about medicines questionnaire: the development and evaluation of a new method for assessing the cognitive representation of medication. Psychol Health.

[14] Azarkeivan A, Mohammadnezhad Gh, Esmaily H (2022). Development of Thalassemia Medication Questionnaire (TMQ): an instrument for Measuring Major Thalassemia patients’ knowledge and practice regarding their medications. J Pharm Care.

[15] Azarkeivan A, Hajibeigi B, Alavian SM, Lankarani MM, Assari S (2009). Associates of poor physical and mental health-related quality of life in beta thalassemia-major/intermedia. J Res Med Sciences: Official J Isfahan Univ Med Sci.

[16] Verma IC, Saxena R, Kohli S (2011). Past, present & future scenario of thalassaemic care & control in India. Indian J Med Res.

[17] Langlois S, Ford JC, Chitayat D (2008). Carrier screening for Thalassemia and hemoglobinopathies in Canada. J Obstet Gynecol Canada: JOGC = J D’obstetrique et gynecologie du Can : JOGC.

[18] Caro JJ, Ward A, Green TC, Huybrechts K, Arana A, Wait S (2002). Impact of Thalassemia major on patients and their families. Acta Haematol.

[19] Kourorian Z, Azarkeivan A, Hajibeigi B, Oshidari A, Shirkavnd A (2014). The effect of knowledge, attitude and practice on the function of thalassemic patients. Iran J Blood Cancer.

[20] Farhud DD, Sadighi H. Investigation of prevalence of Beta-Thalassemia in Iranian provinces. Iran J Public Health. 1;26(1–2):1–6.

[21] Patterson S, Singleton A, Branscomb J, Nsonwu V, Spratling R (2022). Transfusion complications in Thalassemia: patient knowledge and perspectives. Front Med (Lausanne).

[22] Mohammadnezhad G, Sattarpour M, Moradi N (2022). Budget impact analysis of breast cancer medications: a systematic review. J Pharm Policy Pract.

[23] DR. KALPANA B MBGPDKDA. Current and Future Therapies in Thalassemia: Beginning of a New Era. Annals of RSCB [Internet]. 2021Jun.9 [cited 2024May20];25(6):9672-8. http://annalsofrscb.ro/index.php/journal/article/view/7302

