# Supplementary material for: Awareness and practical evaluation of correct use of iron chelators; a study to track the ambiguities of thalassemia patients on their medications in Iran
Source: BMC Res Notes. 2024 Jun 13;17:163. doi: 10.1186/s13104-024-06819-3 (PMC11170868; doi:10.1186/s13104-024-06819-3)
Supplement: Supplementary file 1 — Supplementary Material 1 [file 13104_2024_6819_MOESM1_ESM.pdf]

This article was downloaded by: [Dalhousie University]
On: 10 November 2014, At: 12:27
Publisher: Routledge
Informa Ltd Registered in England and Wales Registered Number: 1072954 Registered office: Mortimer House, 37-41 Mortimer Street, London W1T 3JH, UK

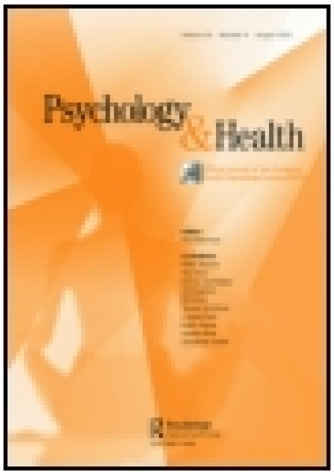

Psychology & Health
Publication details, including instructions for authors and subscription information:
http://www.tandfonline.com/loi/gpsh20

The beliefs about medicines questionnaire: The development and evaluation of a new method for assessing the cognitive representation of medication

Robert Horne [a], John Weinman [b] & Maittew Hankins [c d]

[a] Department of Pharmacy, University of Brighton, Lewes Road, Brighton, BN2 4GJ, UK

[b] Unit of Psychology, United Medical and Dental Schools of Guy's and St Thomas's Hospitals, London, SE1 9RT, UK

[c] Department of Pharmacy, University of Brighton

[d] Division of Psychiatry, United Medical and Dental Schools of Guy's and St Thomas's Hospitals, London, SE1 9RT, UK

Published online: 19 Dec 2007.

To cite this article: Robert Horne, John Weinman & Maittew Hankins (1999) The beliefs about medicines questionnaire: The development and evaluation of a new method for assessing the cognitive representation of medication, Psychology & Health, 14:1, 1-24, DOI: 10.1080/08870449908407311

To link to this article: http://dx.doi.org/10.1080/08870449908407311

PLEASE SCROLL DOWN FOR ARTICLE

Taylor & Francis makes every effort to ensure the accuracy of all the information (the "Content") contained in the publications on our platform. However, Taylor & Francis, our agents, and our licensors make no representations or warranties whatsoever as to the accuracy, completeness, or suitability for any purpose of the Content. Any opinions and views expressed in this publication are the opinions and views of the authors, and are not the views of or endorsed by Taylor & Francis. The accuracy of the Content should not be relied upon and should be independently verified with primary sources of information. Taylor and Francis shall not be liable for any losses, actions, claims, proceedings, demands, costs, expenses, damages, and other liabilities whatsoever or howsoever caused arising directly or indirectly in connection with, in relation to or arising out of the use of the Content.

This article may be used for research, teaching, and private study purposes. Any substantial or systematic reproduction, redistribution, reselling, loan, sub-licensing, systematic supply, or distribution in any form to anyone is expressly forbidden. Terms & Conditions of access and use can be found at http://www.tandfonline.com/page/terms-and-conditions

Psychology and Health, 1999, Vol. 14, pp. 1–24
Reprints available directly from the Publisher
Photocopying permitted by license only

© 1999 OPA (Overseas Publishers Association) N.V.
Published by license under
the Harwood Academic Publishers imprint,
part of The Gordon and Breach Publishing Group.
Printed in Malaysia.

THE BELIEFS ABOUT MEDICINES QUESTIONNAIRE: THE DEVELOPMENT AND EVALUATION OF A NEW METHOD FOR ASSESSING THE COGNITIVE REPRESENTATION OF MEDICATION

ROBERT HORNE[1,*], JOHN WEINMAN[2] and MATTHEW HANKINS[3]

[1]*Department of Pharmacy, University of Brighton, Lewes Road, Brighton BN2 4GJ, UK*
[2]*Unit of Psychology, United Medical and Dental Schools of Guy's and St Thomas's Hospitals, London SE1 9RT, UK*
[3]*Department of Pharmacy, University of Brighton and Division of Psychiatry, United Medical and Dental Schools of Guy's and St Thomas's Hospitals, London SE1 9RT, UK*

(Received 4 August, 1996; in final form 16 July, 1997)

This paper presents a novel method for assessing cognitive representations of medication: the Beliefs about Medicines Questionnaire (BMQ). The BMQ comprises two sections: the BMQ-Specific which assesses representations of medication prescribed for personal use and the BMQ-General which assesses beliefs about medicines in general. The pool of test items was derived from themes identified in published studies and from interviews with chronically ill patients. Principal Component Analysis (PCA) of the test items resulted in a logically coherent, 18 item, 4-factor structure which was stable across various illness groups. The BMQ-Specific comprises two 5-item factors assessing beliefs about the necessity of prescribed medication (*Specific-Necessity*) and concerns about prescribed medication based on beliefs about the danger of dependence and long-term toxicity and the disruptive effects of medication (*Specific-Concerns*). The BMQ-General comprises two 4-item factors assessing beliefs that medicines are harmful, addictive, poisons which should not be taken continuously (*General-Harm*) and that medicines are overused by doctors (*General-Overuse*). The two sections of the BMQ can be used in combination or separately. The paper describes the development of the BMQ scales and presents data supporting their reliability and their criterion-related and discriminant validity.

KEY WORDS: Medicines, attitudes, personal models, illness perceptions, drug therapy, treatment adherence.

INTRODUCTION

The prescription of a medicine is the most common treatment intervention and accounts for the largest single commodity source of health expenditure in most developed economies. However, it is estimated that approximately 30–50% of prescribed medication is not taken as directed (Meichenbaum and Turk, 1987) and non-adherence to medication is seen as a significant challenge to research and practice within the health care domain (Horne, 1993; Horwitz and Horwitz, 1993). Various social cognition models (SCMs) such as the Health Belief Model (HBM: Rosenstock, 1974), the Theory of Reasoned Action (TRA: Ajzen and Fishbein, 1980) and its revision the Theory of

* Corresponding author.

Downloaded by [Dalhousie University] at 12:27 10 November 2014

Planned Behaviour (TPB: Azjen, 1985), have been used to explain variation in medication adherence. This research shows that medication non-adherence may be the result of a rational decision by the patient and identifies some of the cognitions which are salient to these decisions. Although the specific type of beliefs which are associated with adherence varies across studies, certain cognitive variables included in SCMs appear to be prerequisites of adherence in some situations (Horne and Weinman, 1998). For example, beliefs that failure to take the treatment could result in adverse consequences and that one is personally susceptible to these effects tends to be associated with higher adherence rates (e.g. Cummings *et al.*, 1981; Kelly *et al.*, 1987). Additionally, adherence decisions may be influenced by a cost-benefit analysis in which the benefits of treatment are weighted against the perceived barriers (e.g. Brownlee-Duffeck *et al.*, 1987; Cummings *et al.*, 1981). Other studies, based on the TRA/TPB have shown that the perceived views of significant others such as family, friends and doctors (normative beliefs) may also influence adherence (Cochran and Gitlin, 1988; Ried and Christensen, 1988; Ried *et al.*, 1985).

Leventhal's self-regulatory model of illness (SRM) (Leventhal *et al.*, 1980; Leventhal and Cameron, 1987) has also been applied to the study of medication adherence. In the SRM the decision about whether or not to take medication is conceptualised as one of a number of possible procedures for coping with an illness threat (Leventhal *et al.*, 1997). Adherence will be more likely if the patient perceives that the advice to take medication makes 'common-sense', in the light of their experiences (e.g. past illness and/or current symptoms) and their personal beliefs about the illness (Leventhal *et al.*, 1992). In addition to providing an explanatory framework for how beliefs and behaviour are related, self-regulatory theory postulates the types of beliefs which underpin illness cognitions suggesting that the selection of a coping procedure, e.g. to seek (or not to seek) medical advice or to take (or not to take) medication, is guided by beliefs about the nature, duration, causes, consequences and potential for cure/control of the illness.

It has been suggested that representations of *treatment* may also play a role in self-regulation and that the explanatory power of SCMs in relation to medication adherence may be enhanced by assessing patients' beliefs about medication. Decisions about taking medication are likely to be informed by beliefs about medicines as well as beliefs about the illness which the medication is intended to treat or prevent (Horne, 1997). This principle is recognised in a recent report from the Royal Pharmaceutical Society of Great Britain which has identified the role of medication beliefs in treatment adherence as a priority for future research (Marinker, 1997; Royal Pharmaceutical Society of Great Britain, 1997).

Several qualitative studies have shown that people have beliefs about medicines in general (e.g. Britten, 1994; Fallsberg, 1991 and Lorish *et al.*, 1990), as well as beliefs about medication prescribed for specific illnesses such as epilepsy (Conrad, 1985) and hypertension (Morgan and Watkins, 1988). Moreover, certain representations of medicines appear to be common across several illness and cultural groups. However, a systematic comparison of findings is hampered by the fact that the few studies which have quantitatively assessed medication beliefs have used different questionnaires (Woller *et al.*, 1993; Echabe *et al.*, 1992) or have investigated medication beliefs in the broader context of views about the practice of medicine (Marteau, 1990). Furthermore, some studies have assessed peoples' ideas about medicines in general (General beliefs) whereas others have focused on specific medication prescribed for a particular illness (Specific beliefs).

A review of the existing literature on lay beliefs about medicines raises three key questions (Horne, 1997). The first relates to the nature of medication beliefs and whether the

Downloaded by [Dalhousie University] at 12:27 10 November 2014

range of specific and general medication beliefs can be summarised into 'common themes' which are relevant across illness and cultural groups. A second question relates to the distribution of these beliefs (who holds them and how strongly are they held?). Finally, there is the question of how representations of medicine relate to each other (e.g. general vs. specific) and to illness beliefs, as well as to adherence behaviours. We believe that there is need for a psychometrically sound method for operationalising and scoring commonly held beliefs about medication in order to systematically address the above questions. This paper describes the development of a questionnaire-based method for assessing beliefs about Specific and General medication, the Beliefs about Medicines Questionnaire (Section 1) and presents a preliminary evaluation of its psychometric properties (Section 2).

SECTION 1: DEVELOPMENT OF THE BELIEFS ABOUT MEDICINES QUESTIONNAIRE (BMQ)

PARTICIPANTS

A Chronic Illness sample ($n=524$), comprising asthmatic, diabetic and psychiatric patients from hospital clinics and cardiac, general medical and renal (haemodialysis recipients) in-patients. The six illness groups from which patients were sampled were chosen to reflect a variety of disease and treatment characteristics. Patients were included if they had been prescribed one or more medicines for regular use in the treatment of their illness for at least two months prior to the study and if they could read and understand the questionnaire and felt well enough to complete it. Ethical committee approval was granted for the study in each of the participating clinics and hospitals. The characteristics of the main sample are shown in Table 1 and the individual illness samples are described in more detail below.

The Asthmatic sample ($n=78$) comprised consecutive asthmatic patients attending the weekly out-patient clinics of two consultant respiratory physicians at a general hospital in

Table 1 Demographic characteristics of the chronic illness sample

	Asthma clinic	*Diabetic clinic*	*Renal dialysis IP*	*Cardiac IP*	*Psychiatric clinic*	*General medical IP*
n	78	99	47	120	89	91
Gender (% male)	37	39	49	71	37	50
Age (mean, SD)	45.5 (18.3)	46.6 (18.5)	49 (17.3)	63.6 (12.4)	45.8 (10.9)	54 (19.8)
Educational experience		*				
Secondary (%)	68.0		59.6	81.4	47.8	71.8
Tertiary (%)	22.7		21.3	11.5	28.4	23.5
Advanced (%)	9.3		19.1	7.1	23.8	4.7
Number of prescribed medicines (mean, SD)	3.5 (1.7)α	#	7.1 (1.9)β	3.5 (2.3)β	2.2 (1.4)α	4.1 (3.2)β

*Data unavailable.
IP = Hospital In-patient.
α = Patient report of number of prescribed medications.
β = Number of prescribed medication obtained from the patient's medical notes.
#The exact number of medicines prescribed for each patient was not recorded. However the majority of patients were prescribed only one medication (Insulin or a single oral anti-hypoglycaemic agent).

Downloaded by [Dalhousie University] at 12:27 10 November 2014

Brighton, UK, during a 3-month period between March and May 1994. Of 105 asthmatic patients on the clinic schedule 17 refused to take part in the study, 9 did not attend the clinic and one who agreed to take part subsequently withdrew without completing the questionnaire. Seventy-eight patients entered the study and completed the clinic questionnaire giving an overall response rate 78/105 = 74.3%. The mean duration of asthma was 1.6 years (SD = 1.3).

The Diabetic sample ($n = 99$) comprised consecutive attenders at a diabetic out-patient review clinic in a London general hospital. In a six week period, during April and May 1994, 124 study-eligible patients were approached and 20 refused to take part. Five of the 104 questionnaires returned were rejected (> 10% of responses to questionnaire statements were missing or illegible) giving a final completion rate of 79.8%. Sixty four (64.7%) of the patients were insulin-treated while the remaining patients received oral hypoglycaemic medication.

The Renal sample ($n = 47$) was recruited from the renal unit at a London Teaching Hospital. Patients were randomly selected from the dialysis list and evaluated for entry into the study until a target sample of approximately half of the 103 patients on the hospital haemodialysis list were recruited. Of 59 randomly selected study-eligible patients, 47 agreed to take part and completed the questionnaire giving a response rate of 79.7%. The mean duration of dialysis treatment was 4.5 years (SD = 4.9).

The Psychiatric sample ($n = 89$) was recruited as part of an audit evaluating a medicines-information service at a hospital psychiatric out-patient clinic in Brighton, UK. Of 118 patients who were eligible for inclusion in the present study, 27 failed to attend the 'research clinic' and a further two patients were omitted because they did not legibly complete over 90% of the questionnaire items. The final study sample therefore comprised 89 patients giving a response rate of 78.4%. The mean duration of psychiatric illness was 10.2 years (SD = 8.4).

The Cardiac and General Medical inpatient samples ($n = 120$; $n = 91$ respectively) were recruited from general medical wards of two London teaching hospitals and five district general hospitals in London and Brighton, over an 8 week period between January and March, 1995. Of 254 study-eligible patients, 37 refused to take part and 217 entered in the study. Six of the questionnaires were rejected (> 10% of responses to questionnaire statements were missing or illegible). The remaining 211 questionnaires were retained for analysis. The final completion rate was therefore 211/254 = 83.1%. On the basis of primary diagnosis the sample comprised chronic cardiac disease (56.8%), chronic respiratory diseases (16.2%), gastro-intestinal disorders (10.9%), diabetes (9.9%), cancer (3.8%) and epilepsy (2.4%). Patients with chronic cardiac disease were considered as a single illness group and the remaining patients ($n = 91$) were grouped together as the 'General medical inpatients'.

METHOD

Rationale and Overview

The BMQ was intended to assess commonly-held beliefs about medicines. The primary task was to simplify the fairly broad range of beliefs which people hold about Specific and General medication into 'core themes' which could then be evaluated as psychometric scales. The BMQ scales were derived from a pool of items representing commonly held beliefs about medication (see below for details) using exploratory Principal

Downloaded by [Dalhousie University] at 12:27 10 November 2014

Components Analysis (PCA). Specific and General medication beliefs were analysed separately. The factor structures obtained were then tested in three ways. Confirmatory factor analysis (Tabachnick and Fidell, 1993) was used to verify the factor structure. The stability of the factor structure across chronic illness groups was tested by investigating whether the factor structure obtained by exploratory PCA in one illness group was replicated in other illness groups. Finally, to confirm the validity of separating Specific and General medication beliefs, items loading on the Specific and General factors identified by PCA were combined and subjected to a further PCA. A high degree of separation between general and specific items would indicate that patients made clear distinctions between specific and general medication and justify the division of the BMQ into Specific and General components.

Item Pool

A pool of 34 statements representing commonly held beliefs about specific ($n = 16$) and general medication ($n = 18$) was obtained by selecting beliefs identified in the literature which appeared to be common to patients with a range of chronic illnesses and from interviews we conducted with 35 patients receiving regular medication for chronic illness (20 haemodialysis patients and 15 patients with myocardial infarction). In these interviews patients were asked open questions eliciting their views about medicines prescribed for them and their thoughts about medicines in general in an attempt to identify common beliefs which had not emerged in previous studies. The final pool of 34 items, together with their origin, is shown in Table 2. Twelve items were positive statements about medicines (e.g. '*Without medicines doctors would be less able to cure people*') and the remaining 22 items focused on negative (e.g. '*Most medicines are addictive*') or neutral aspects (e.g. '*Medicines only work if they are taken regularly*'). This balance of items reflects that observed in the literature (e.g. Britten, 1994; Donovan and Blake, 1992; Fallsberg, 1991 and Lorish *et al.*, 1990; Morgan and Watkins, 1988; Conrad, 1985) and in interviews with patients. Responses to each statement were scored on a 5-point Likert scale (where 1 = strongly disagree, 2 = disagree, 3 = uncertain, 4 = strongly agree and 5 = strongly agree) and subjected to PCA as described below. Although the psychiatric and diabetic samples received identical General items as the other illness groups, the Specific item pool differed by one item in the case of the psychiatric sample and two items for the diabetic sample. This was done in order to reflect issues which were perceived to be pertinent to these groups. For the psychiatric sample the item '*Without my medicines I would be very ill*' was replaced by '*Only my medicines can control my mental health problems*'. For the diabetic sample the items '*My life would be impossible without my medicines*' and '*My medicines protect me from becoming worse*' were replaced by the items '*My medication controls my diabetes*' and '*My medication prevents my blood sugar from becoming too high*'. For this reason, the derivation of the BMQ-Specific scales was based on data from the asthmatic, cardiac, renal and general medical in-patient groups which had received identical Specific items.

PROCEDURE

Each participant was invited to take part in a study of patients' views about their illness and treatment. The investigators stressed that the study was being conducted by the

Downloaded by [Dalhousie University] at 12:27 10 November 2014

Table 2 Pool of medication statements subjected to PCA including details of source

	Source
Statements about specific medication prescribed for the patient	
My health, at present, depends on my medicines	1
Having to take medicines worries me	2,3,4
My life would be impossible without my medicines	1
My medicines are powerful	1
Without my medicines I would be very ill	1
I sometimes worry about the long-term effects of my medicines	3,5
My medicines are a mystery to me	1
My medicines are effective	1
My medicines disrupt my life	2,6,7
I sometimes worry about becoming too dependent on my medicines	2,3,8
My health in the future will depend on my medicines	1,9
My medicines protect me from becoming worse	1,2
I would like to change my present treatment	1
It is difficult for me to take my medicines in exactly the way my doctor told me	1
I can cope without my medicines	1,2
I am in control of my medication	2,10
Statements about medicines in general	
Without medicines doctors would be less able to cure people	1
Newer medicines are more effective than older ones	1
Most medicines are addictive	3
People who take medicines should stop their treatment for a while every now and again	2,3
Medicines only work if they are taken regularly	1
Medicines do more harm than good	5
Medicines are not natural remedies	1,3,4,8,11,12
All medicines are poisons	4
It is better to do without medicines	1,2,4,8,
Natural remedies are safer than medicines	1,3,4,8,11,12,13
Stronger medicines are more dangerous than weaker medicines	14,15
Medicines are a necessary evil	4,13
Doctors place too much trust on medicines	1,16
If doctors had more time with patients they would prescribe fewer medicines	1,16
There is a big difference between a medicine and drug	13,16
The medicine you get is more important than the doctor you see	1
Doctors use too many medicines	1,5,16
Most medicines are safe	1

Source of statements

1. Interviews conducted with 35 chronically ill patients
2. Conrad, 1985
3. Morgan and Watkins, 1988
4. Fallsberg, 1991
5. Clinthrone *et al.*, 1986
6. Becker *et al.*, 1978
7. Cochran and Gitlin, 1988
8. Donovan and Blake, 1992
9. Arluke, 1980
10. Helman, 1988
11. Coulter, 1985
12. New and Senior, 1991
13. Gabe and Lipshitz-Phillips, 1982
14. Lorish, 1990
15. Leventhal, 1986
16. Rees-Jones, 1979

University and was completely independent of the hospital and that responses were confidential and anonymous and would not be seen by any of the staff involved in their care. It was hoped that this would encourage participants to respond in a way which represented their own views rather than those which they considered to be socially desirable

Downloaded by [Dalhousie University] at 12:27 10 November 2014

(Abraham and Hampson, 1996) and so avoid any response bias which might have resulted if patients had associated the researcher with the clinical team. Participants were presented with the 34 item pool as described above at the same time as a battery of questionnaires assessing other relevant constructs as described in Section 2 below. These measures (e.g. reported adherence and beliefs about illness) were included to assess the criterion-related validity of the BMQ and were chosen on the basis of hypothesised relations with medication beliefs. The instructions to participants, are shown in the Appendix. Clinic patients were asked to complete the questionnaire while waiting to see the doctor. Patients recruited from hospital wards were asked to complete the study questionnaire by the researcher who then arranged to collect it at a convenient time.

Downloaded by [Dalhousie University] at 12:27 10 November 2014

Principal Component Analysis (PCA)

PCA was conducted using the non-orthogonal (Direct Oblimin) method of rotation as recommended by Kline (1994) and Cattell (1995). Cases with missing data were deleted listwise and items were omitted on the basis of the Kaiser-Meyer-Olkin (KMO) statistic for each item (item omitted if KMO < 0.7), factor scree plot and final factor loading as recommended by Norusis (1992). In order to eliminate the influence of multi-dimensional outliers, items retained within the final factor structure were "cleaned" by removal of multivariate outliers (Mahalanobis distance > 3 standard deviations from the mean) and removal of cases with greater than five missing items (Tabachnick and Fidell, 1993).

Selecting Items for the BMQ Scales Using Exploratory PCA

The exploratory PCA of Specific beliefs about medicines prescribed for personal use was performed on responses to the 16 items representing beliefs about prescribed medication (*Specific*), shown in Table 2 above. The responses from the cardiac sample ($n = 120$) were analysed first. The rationale for choosing a single diagnostic group was that patients with one illness might receive very different medication from those with another and this might influence representational structures. We could not assume that patients with different illnesses would have similar ideas about their medication. Rather, our goal was to identify a simple factor structure for a single diagnostic group and then to test whether this structure was stable across other illness groups. The cardiac sample was chosen for initial analysis on the grounds that it was the single largest diagnostic group within the main sample.

The rationale for limiting initial exploratory factor analysis of specific items to a single illness group did not apply to beliefs about medicines in general. Here, the aim was to explore representations of medication as a broad concept, rather than beliefs which might be unique to a particular illness group. In an attempt to obtain a factor structure which was representative of patients with a range of chronic illnesses, data obtained from three diagnostic groups (asthmatic, diabetic and renal) were amalgamated and subjected to an exploratory PCA. The reason for selecting these particular diagnostic groups for combination was that the cardiac and general hospital samples were derived from the same population of hospital in-patients. Data were combined in order to investigate the themes underlying beliefs about medicines in general which would be common across chronic illness populations. Thus combining the cardiac and general medical inpatient samples may have reduced the 'scope' of the sample.

Testing the Factor Structure Derived from Exploratory PCA

Confirmatory factor analysis was performed by computing Pearson's correlations for factor loadings against a theoretical model of the predicted factor loadings (Tabachnick and Fidell, 1993). The theoretical model was defined by assigning a factor a loading of '1' to all items expected to load on the factor. All other items were assigned a loading of '0'. In this way, the expected pattern of loadings could be compared with that derived from the comparison groups.

The stability of the factor structure obtained for Specific beliefs in the cardiac group was tested by a further series of PCA on the responses to factor items obtained from the asthmatic, renal and general medical inpatient samples. The stability of the factor structure for General medication beliefs obtained from the amalgamated data set (asthmatic, diabetic, renal samples) was tested by investigating the extent to which the structure could be replicated when the factor items were entered in 3 separate PCAs using data from the individual cardiac, general medical and psychiatric samples.

The separation of Specific and General items was tested by a further PCA of the combined items loading on the factors identified by exploratory PCA. This analysis was performed on pooled data from all six illness groups ($n = 524$). PCA was performed using non-orthogonal (Direct Oblimin) rotation and setting a 4-factor solution as suggested by factor scree plot.

RESULTS

Exploratory PCA

Specific beliefs. The mean and SD for each of the 16 items eliciting beliefs about prescribed medication administered to the Cardiac sample are shown in Table 3.

Four items with KMO values < 0.7 were omitted. Factor scree plot analysis suggested a 2-factor solution explaining 51% of the variance. Having arrived at a core structure of two 5-item factors the data set was cleaned by removal of multivariate outliers (Mahalanobis distance > 3 standard deviations from the multi-dimensional mean) and removal of cases with greater than five missing items. This resulted in omission of 6 cases. Re-factoring on the 114 remaining cases produced a similar two factor structure explaining 53% of the variance.

Factor labels. The final 2-factor structure is shown in Table 4. The first factor comprised items relating to the positive effect of medication on health and were representative of the perceived necessity of medication for maintaining health. This factor was labelled *Specific-Necessity*. The second factor comprised items relating to concerns about the adverse consequences of medication based on beliefs about the potential for dependence or harmful long-term effects and that medication taking is disruptive. This factor was labelled *Specific-Concerns*.

General beliefs. The mean and standard deviation for scores on each of the 18 items eliciting beliefs about medicines in general are shown in Table 3. Elimination of six items with a low KMO statistic (< 0.7) and setting a two factor solution as suggested by scree plot analysis, followed by elimination of a further 4 items with low or diffuse loading resulted in two 4-item factors shown in Table 5.

Downloaded by [Dalhousie University] at 12:27 10 November 2014

Downloaded by [Dalhousie University] at 12:27 10 November 2014

Table 3 Mean and standard deviation SD of responses to specific and general statements

	Mean	*SD*
Statements about prescribed medication (Specific)		
It is difficult for me to take my medicines in exactly the way my doctor told me	2.09	0.75
My medicines disrupt my life	2.31	0.92
Having to take medicines worries me	2.70	1.07
I sometimes worry about becoming too dependent on my medicines	2.82	1.10
My medicines are a mystery to me	3.00	0.98
I sometimes worry about the long-term effects of my medicines	3.11	1.15
My medicines are powerful	3.33	0.77
I would like to change my present treatment	3.44	1.01
My life would be impossible without my medicines	3.51	0.95
My health in the future will depend on my medicines	3.62	0.93
I can cope without my medicines	3.62	0.96
Without my medicines I would be very ill	3.66	0.88
I am in control of my medication	3.73	0.85
My medicines protect me from becoming worse	3.91	0.71
My medicines are effective	3.94	0.56
My health, at present, depends on my medicines	4.03	0.73
Statements about medicines in general (General)		
Without medicines doctors would be less able to cure people	3.13	1.54
Newer medicines are more effective than older ones	3.37	0.84
Most medicines are addictive	2.73	0.89
People who take medicines should stop their treatment for a while every now and again	2.54	0.91
Medicines only work if they are taken regulary	3.75	0.80
Medicines do more harm than good	2.24	0.85
Medicines are not natural remedies	3.13	0.92
All medicines are poisons	2.24	0.97
It is better to do without medicines	2.61	1.08
Natural remedies are safer than medicines	2.88	0.91
Stronger medicines are more dangerous than weaker medicines	3.24	0.90
Medicines are a necessary evil	3.06	1.10
Doctors place too much trust in medicines	2.90	0.93
If doctors had more time with patients they would prescribe fewer medicines	3.17	0.98
There is a big difference between a medicine and a drug	3.24	0.88
The medicine you get is more important than the doctor you see	2.87	1.14
Doctors use too many medicines	2.84	0.91
Most medicines are safe	2.72	0.92

The first factor comprised items expressing beliefs about the way in which medicines are used by doctors. The essence of this factor, labelled *General-Overuse* is the notion that medicines are over-prescribed by doctors who place too much trust in them. The second factor, labelled *General-Harm* concerns the potential of medication to harm and comprises representations of medication as harmful, addictive, poisons and the belief that people who take medicines should stop their treatment every now again.

Testing the Factor Structure

Confirmatory factor analysis. The results for the BMQ-General and BMQ-Specific factor structures are presented in Tables 6 and 7.

Table 4 Factor structure obtained by principal components analysis of BMQ-Specific items ($n = 114$* patients with chronic heart diseases)

Structure Matrix: Principal components analysis with non-orthogonal (Direct Oblimin) rotation.	*Factor 1 Specific-Necessity*	*Factor 2 Specific-Concerns*
My life would be impossible without my medicines	0.81	−0.06
Without my medicines I would be very ill	0.78	0.09
My health, at present, depends on my medicines	0.71	−0.02
My medicines protect me from becoming worse	0.67	−0.19
My health in the future will depend on my medicines	0.62	−0.11
I sometimes worry about the long term effects of my medicines	−0.00	0.80
Having to take my medicines worries me	−0.18	0.78
I sometimes worry about becoming too dependent on my medicines	−0.19	0.72
My medicines disrupt my life	0.05	0.67
My medicines are a mystery to me	−0.00	0.58
Eigenvalue	2.8	2.4
Percentage variance explained	28.5	24.0

*6 cases were removed during the cleaning procedure.

Table 5 Factor structure obtained by PCA of BMQ-General items ($n = 219$ patients with chronic illnesses-asthmatic = 77, diabetic = 99, haemodialysis recipients = 42)*

Structure Matrix: Principal components analysis with non-orthogonal (Direct Oblimin) rotation.	*Factor 1 General-Overuse*	*Factor 2 General-Harm*
If doctors had more time with patients, they would prescribe fewer medicines	0.80	0.11
Doctors use too many medicines	0.79	0.15
Doctors place too much trust in medicines	0.72	0.24
Natural remedies are safer than medicines	0.70	0.33
Medicines do more harm than good	0.33	0.72
People who take medicines should stop their treatment for a while every now and again	0.18	0.70
Most medicines are addictive	0.02	0.70
All medicines are poisons	0.28	0.69
Eigenvalue	2.8	1.5
Percentage variance explained	35.3	19.0

*Five cases were removed during the cleaning procedure.

Table 6 Confirmatory factor analysis for BMQ-General

	Pearson correlation of items with predicted factor pattern		
	Cardiac	*Psychiatric*	*General medical*
General-Overuse	0.90	0.88	0.70
General-Harm	0.93	0.83	0.73

Downloaded by [Dalhousie University] at 12:27 10 November 2014

Table 7 Confirmatory factor analysis for BMQ-General and BMQ-Specific scales

	Pearson correlation of items with predicted factor pattern					
	Cardiac	*Asthma*	*Renal*	*General medical*	*Psychiatric*	*Diabetes*
BMQ-General						
Overuse	0.90	NA	NA	0.70	0.88	NA
Harm	0.93	NA	NA	0.73	0.83	NA
BMQ-Specific						
Necessity	0.98	0.92	0.88	0.95	0.83	0.90
Concerns	0.98	0.88	0.88	0.90	0.96	0.95

Downloaded by [Dalhousie University] at 12:27 10 November 2014

Replication of factor structure. The 2-factor structure for Specific beliefs was replicated by PCA of the responses to the 10 items obtained from asthmatic, renal and general medical inpatient samples. Although there were minor differences in factor loadings, the factor structure obtained for each of the samples contained identical items. The 2-factor structure obtained for General beliefs by exploratory PCA of combined data from the asthmatic, diabetic and renal samples was replicated in the cardiac, and psychiatric samples, indicating acceptable stability of the factor structures across illness groups. PCA of the data from the General Medical in-patients, produced a similar factor structure, with the exception of one item: "*Natural remedies are safer than medicines*" which had migrated from factor 1 to factor 2.

PCA of combined Specific and General factor items. PCA of pooled data from all 6 illness samples showed a clear separation of Specific and General items. A 4-factor structure was obtained (see Table 8) which closely resembled the original Specific and General factor structures except that one item from the *Specific-Concerns* factor '*My medicines are a mystery to me*', loaded a little higher on the *General Harm* (0.55) than on *Specific-Concerns* (0.39). Removal of the General Medical Inpatient sample from the data set followed by a further PCA on pooled data from the discreet diagnostic groups (asthmatic, diabetic, renal, cardiac and psychiatric) replicated the original Specific and General factor structures.

SECTION 2: EVALUATION OF THE PSYCHOMETRIC PROPERTIES OF THE BMQ

PARTICIPANTS

1. The Chronic Illness sample ($n = 524$), described in Section 1 above.
2. A matched group of patients seeking care from allopathic (community pharmacy) and complimentary sources (homeopathy/herbal clinic). This sample was recruited in order to compare medication beliefs of allopathic and complementary care seekers. The Allopathic Care sample were recruited from a community pharmacy during weekday evenings over a four week period between January and February 1996. Consecutive patients presenting a prescription at a community pharmacy were approached by the researcher while they were waiting for the prescription to be dispensed. One hundred and twenty six study-eligible patients were approached, 22 refused to take part and 104 patients entered the study and returned completed questionnaires

Downloaded by [Dalhousie University] at 12:27 10 November 2014

Table 8 Structure matrix obtained by PCA on combined items from the Specific and General medication belief factors on pooled data from the six illness groups comprising the main sample (total $n = 524$)

	Item *S refers to medicines prescribed for a specific illness* *G refers to medicines in general*	*Factor 1 Specific Concerns*	*Factor 2 Specific Necessity*	*Factor 3 General Harm*	*Factor 4 General Overuse*
S	Having to take this medicine worries me	0.80	0.07	0.15	0.19
S	I sometimes worry about becoming too dependent on my medicines	0.78	−0.02	0.14	0.20
S	I sometimes worry about the long term effects of my medicines	0.76	0.07	0.17	0.15
S	My medicines disrupt my life	0.60	0.16	−0.06	0.33
S	My life would be impossible without medicines	0.12	0.81	−0.07	0.01
S	My health, at present, depends on medicines	0.10	0.76	−0.04	−0.04
S	Without medicines I would be very ill	0.17	0.74	−0.08	0.11
S	My health, in the future, will depend on medicines	0.00	0.70	−0.09	−0.01
S	My medicines protect me from becoming worse	−0.11	0.65	−0.22	−0.04
G	If doctors had more time they would prescribe fewer medicines	0.16	−0.10	0.81	0.09
G	Doctors place too much trust in medicines	0.04	−0.10	0.75	0.23
G	Doctors use too many medicines	0.26	−0.13	0.71	0.17
G	Natural remedies are safer than medicines	0.01	−0.12	0.47	0.45
G	Most medicines are addictive	0.07	0.06	0.05	0.71
G	Medicines do more harm than good	0.22	−0.11	0.22	0.67
G	All medicines are poisons	0.16	0.14	0.21	0.58
S	My medicines are a mystery to me	0.39	0.00	−0.09	0.55
G	People who take medicines should stop their treatment for a while every now and again	0.33	−0.12	0.20	0.51
	Eigenvalue	3.38	2.92	1.60	1.44
	Percentage variance explained	18.8	16.2	8.9	8.0
	Cumulative percentage variance explained	18.8	35.0	43.9	51.9

(>90% items answered legibly). The response rate for the Allopathic Care sample was therefore 104/126 = 83%. The Complementary Care sample were recruited from the clinics of a single herbalist and single homeopath, in Brighton, during the same time period as the Allopathic Care sample. Both practitioners felt that it would be inappropriate to base a researcher in the clinic and so patients were invited to take part in the study by the herbalist/homeopath. Those who agreed were asked to fill out the questionnaire and return it to the author at the University of Brighton in the stamped addressed envelope provided. Fifty-four questionnaires were given out and 36 completed questionnaires were returned. The final response rate for the Complementary Care sample was therefore 36/53 = 67.9%.

Matched samples. Seventy two participants were matched for age and sex and educational experience. Patients from the Allopathic Care sample were selected to match the age and gender profile of the Complementary Care group. Matching was carried out because of the large disparity in group sizes and the possible confounding effect of age and gender. The characteristics of the matched samples are shown in Table 9.

There were no significant differences between Allopathic and Complementary samples in terms of age, and gender. The Complementary Sample had significantly greater educational experience (Pearson Chi-Square = 6.34; DF = 2; $p < 0.05$) and had made significantly more visits to homeopathic ($t = 3.35$; $n = 72$; $p < 0.001$) and herbal ($t = 4.84$; $n = 72$; $p < 0.001$)

Table 9 Characteristics of the Auxiliary Sample (a matched sample of recipients of Allopathic and Complimentary Care)

	Allopathic care sample	*Complementary care sample*
n	36	36
Age [mean (SD)]	42.3 (11.1)	47.3 (18.6)
Gender: number (%) male	9 (25)	8 (22)
Educational Experience		
Secondary (%)	66.6	44.4
Tertiary (%)	16.7	16.7
Advanced (%)	16.7	38.9
Mean (SD) number of visits over previous 6 months to:		
• General practitioner	2 (1.8)	1.7 (1.9)
• Homeopath	0.03 (0.17)	0.78 (1.33)
• Herbalist	0	1.5 (1.9)
Mean (SD) Number of hospital admissions over previous year	0.36 (1.1)	0.19 (0.58)

practitioners in the 6 months prior to the study than had the Allopathic Care sample. There were no significant differences between the samples in the number of reported visits to NHS General Practitioners or hospital admissions. The latter finding was interpreted as an indicator that the samples were comparable in terms of illness severity.

MEASURES

- *The Illness Perception Questionnaire (IPQ) (Weinman et al., 1996).* The IPQ comprises five scales measuring the five components of illness representation specified in Leventhal's self-regulatory model of illness (Leventhal *et al.*, 1980). The five scales assess *identity* (the symptoms the patient associates with the illness), *cause* (personal ideas about aetiology), *time line* (the perceived duration of the illness), *consequences* (expected effects and outcome), and *curelcontrol* (beliefs about potential for cure and control of the illness). The psychometric properties of the IPQ have been evaluated in 7 patient groups including asthmatic, diabetic and hospital haemodialysis recipients and the internal consistency, test-retest reliability and the concurrent, discriminant and predictive validity of the IPQ scales are within acceptable limits (Weinman *et al.*, 1996).
- *Reported Adherence to Medication (RAM) scale.* Published adherence self-report scales were thought to be unsuitable because they are not specific to medication (DiMatteo *et al.*, 1993; Kravitz *et al.*, 1993) or because they do not elicit self-report of the frequency of adjusting or altering dosages (Morisky, 1986). A reported adherence to medication scale (RAM) was therefore devised for the present study. Non-adherence was indicated by the tendency to forget to take medication and to deliberately adjust or alter the dose from that recommended by the physician. The RAM scale comprises four adherence statements. Two items (*'I sometimes forget to take my medicines'* and *'I sometimes alter the dose of my medication to suit my own needs'*) are scored on a 5-point Likert scale with reverse scoring (where 1 = strongly agree; 2 = agree; 3 = uncertain; 4 = disagree and 5 = strongly disagree). A further two items (*'Some people forget to take their medicines. How often does this happen to you?'* and *'Some people I have talked to say that they miss out a dose of their medication or adjust it to suit their own needs. How often do you do this?'*) are phrased as direct questions asking the patient to report

Downloaded by [Dalhousie University] at 12:27 10 November 2014

Table 10 Items assessing medication-related cognitions used for psychometric evaluation of the BMQ scales

Item statements	*Medication-related cognition which item assess*
Items from original pool (see Table 3) retained for psychometric evaluation	
• *I would like to change my present treatment*	Dissatisfaction with present treatment
• *I can cope without my medicines*	Perceived ability to cope without prescribed medicines
• *It is better to do without medicines*	General reluctance to use medicines
Items not included in the PCA items pool from which the BMQ scales were derived	
• *I have been given enough information about my medicines*	Satisfaction with amount of medicines information received
• *I cannot always trust my medicines*	Lack of trust in prescribed medication

the frequency of adjusting or forgetting medication (scored on a 5-point scale where 5 = never, 4 = rarely, 3 = sometimes, 2 = often and 1 = very often). A total medication adherence score is obtained by summing responses to each of the four individual items. Scores ranged from 4 to 20, with higher scores indicating greater reported adherence. The Cronbach alpha coefficients for the RAM scale in the main sample range from 0.6–0.83.

- *The Sensitive Soma (SS) Scale*. This 5-item scale assesses perceptions of personal sensitivity to the potential adverse effects of medication (e.g. '*Even small amounts of medicines can upset my body*'). The scale is currently under development at Rutgers University New Jersey, USA (Diefenbach *et al.*, 1997) and details of scale items are available from the authors. Responses are scored on a 5-item Likert scale and the individual item scores are summed to give a total *Sensitive Soma* score ranging from 5 to 25 where high scores = high perceived sensitivity to the potential adverse effects of medication: This *Sensitive Soma* scale was administered to the cardiac ($n = 120$) and general medical in-patient ($n = 91$) samples. The internal reliability of the scale, as measured by Cronbach's alpha, was acceptable in both groups (general-medical = 0.80; cardiac = 0.78).
- *Single measures assessing medication-related cognitions*. The psychometric evaluation of the BMQ utilised three of the single item statements from the original 34-item pool described above. The items had not loaded on the BMQ factors and so did not represent a Specific-Necessity, Specific-Concern, General-Harm or General-Overuse cognition. However, they seemed, at face value, to represent interesting medication related cognitions and so were used for psychometric evaluation of the BMQ scales.

In addition to these items a further two single item statements were also included as shown in Table 10. Responses to all five single items were: scored on a 5-point Likert scale where 1 = strongly disagree and 5 = strongly agree.

TESTING THE CRITERION-RELATED AND DISCRIMINANT VALIDITY OF THE BMQ

Criterion-related validity

The assessment of the criterion-related validity of each of the BMQ scales was based on the following predictions:

1. *Specific-Necessity*. Patients with stronger beliefs in the necessity of their medication would be less likely to believe that they can cope without it. Thus scores on the

Downloaded by [Dalhousie University] at 12:27 10 November 2014

Specific-Necessity scale would be negatively correlated with scores on the item: *'I can cope without my medicines'*. Beliefs in the necessity of prescribed medication would also be related to perceptions of illness. In particular, patients who believed that their illness would last a long time and who experienced more symptoms would have stronger beliefs in the necessity of the medication prescribed to treat it. Thus *Specific-Necessity* scores would be positively correlated with scores on the Identity and Timeline components of the IPQ which respectively assess perceptions of symptom severity and likely duration of the illness.

2. *Specific-Concerns*. Patients with stronger concerns about their prescribed medication would be more distrustful of it, would tend to want more information about it and would be more likely to want to change their current treatment. Thus it was hypothesised that the *Specific-Concerns* scale scores would be positively correlated with scores on the 'Lack of trust in prescribed medication' and 'Desire to change present treatment' items and would be negatively correlated with scores on the 'Satisfaction with amount of medicines information received' item. Additionally, those who perceived themselves to be susceptible to the potential adverse effects of medication would have stronger concerns about their prescribed medication. Thus scores on the *Specific-Concerns* scale would be positively correlated with scores on the *Sensitive Soma* scale.
3. *General-Harm*. Patients who believed that medicines in general are intrinsically harmful would be more likely to believe that it is better to avoid taking them. Thus scores on the *General-Harm* scale would be positively correlated with scores on the *'It is better to do without medicines'* and *'I can cope without my medicines'* items. Moreover, participants who believed that medicines in general are intrinsically harmful would be more likely to consider themselves to be susceptible to potential adverse effects of medication. Thus scores on the *General-Harm* scale would be positively correlated with scores on the *Sensitive Soma* scale which assess perceptions of personal sensitivity to the adverse effects of medication.
4. *General-Overuse*. Scores on the *General-Overuse* scale would be positively correlated with scores on the *'I can cope without my medicines'* and the *'It is better to do without medicines'* items.
5. *Relations between BMQ scales and reported adherence to medication (RAM)*. It was hypothesised that stronger beliefs in the necessity of prescribed medication would be associated with higher reported adherence. Thus, *Specific-Necessity* scores would be positively correlated with the RAM scale scores. Conversely, patients with stronger concerns about prescribed medication and those who believed that medicines in general were harmful substances which are overused by doctors would report lower medication adherence rates. Thus correlations between the Specific-Concerns, General-Harm and General-Overuse and the RAM scale would be negative.

Discriminant Validity

The discriminant validity of the BMQ-Specific scales was tested on the basis of their ability to distinguish between different illnesses and hence treatment modalities. The discriminant validity of the BMQ-General scales was tested on the basis of their ability to distinguish between patients presenting a personal prescription at a community pharmacy and those seeking complementary therapies. The specific hypotheses were as follows:

1. *Specific-Necessity*. Beliefs about the necessity of prescribed medication would be influenced by the type of treatment typically prescribed for the illness. The characteristic

Downloaded by [Dalhousie University] at 12:27 10 November 2014

effects of medication on symptoms would be particularly important. For example, diabetic patients who fail to take their treatment may become severely ill very quickly. Asthma medication often produces symptom relief which the patient can clearly relate to taking the medication. Similarly, omitting medication may quickly result in adverse symptoms. Conversely, patients receiving medication for mental health related problems may perceive a much more tenuous link between their medication and concrete benefit in terms on symptoms. Thus it was hypothesised that: *Specific-Necessity* scores would discriminate between patients from different diagnostic groups. In particular, diabetic patients would be expected to have higher scores than asthmatic patients who in turn would have higher mean *Specific-Necessity* scores than psychiatric out-patients.

2. *Specific-Concerns*. Asthma treatment often incorporates corticosteroids. This is a large group of compounds, some of which are associated with adverse side-effects. Additionally, other members of this drug group are frequently misused in sport and have a high "media-profile". Patients' concerns could be influenced by this, particularly if they fail to differentiate between steroids they are taking for asthma (which are generally inhaled and therefore less "dangerous") and the more potent formulations which are often the subject media attention. Similarly, psychiatric out-patients are often prescribed 'tranquillisers', which have also received adverse media attention (Cohen, 1983). Thus it was hypothesised that *Specific-Concerns* scores would discriminate between patients from different diagnostic groups. In particular, asthmatic and psychiatric patients would have higher mean *Specific-Concerns* scores than other illness groups.
3. *General-Harm* and *General-Overuse*. People who believe that medicines in general are intrinsically harmful substances which are overused by doctors may be more inclined to seek alternative methods of treatment. The hypothesis used to test the discriminant validity of the BMQ-General scales was that people seeking care from a homeopathic or herbal clinic would have higher mean scores on the *General-Harm* and *General-Overuse* scales than those presenting a prescription for dispensing by a community pharmacist.

Downloaded by [Dalhousie University] at 12:27 10 November 2014

PROCEDURE

The psychometric evaluation was conducted on the basis of interactions between the BMQ factors and the above measures which had been administered to the main sample at the same time as the pool of mediation belief items from which the BMQ was derived. The Allopathic/Complementary Care samples were recruited after the BMQ had been derived from the main sample (as detailed in Section 1). Only the 8-item BMQ-General (comprising the *General-Overuse* and *General-Harm* scales) was administered to the Allopathic/Complementary Care samples. The *Sensitive Soma* Scale was not available when the asthmatic, diabetic, renal and psychiatric samples were recruited. The scale was however available when the cardiac and general medical samples were recruited a few months later. Thus different samples were used to evaluate different psychometric properties. The internal reliability of each scale was evaluated for all 6 illness groups comprising the main sample. Test-retest reliability was evaluated using the asthmatic sample. Repeat questionnaires were sent to the patients, together with a stamped addressed envelope, two weeks after they had been seen in clinic. Criterion-related validity of the BMQ-Specific scales was evaluated using the asthmatic sample, except for interaction between the *Specific-Concerns* and Sensitive Soma scales which were evaluated using the

general medical inpatient samples. Relations between BMQ scales and RAM were evaluated on pooled data from the Cardiac and General Medical samples. The discriminant validity of the BMQ-Specific scales was evaluated in the main sample. The discriminant validity of the BMQ-General scales was evaluated in the Allopathic/Complementary Care sample.

Statistical Techniques

The internal consistency of each BMQ scale was evaluated using Cronbach's alpha. Spearman correlations (ρ) were used to evaluate test-retest reliabilities between initial and repeated test scores for each scale and also the relations between scales used to test the criterion-related validity of the BMQ. The *a priori* hypotheses relating to the discriminant validity of the BMQ-Specific scales were investigated using one-way ANOVA and linear contrasts. Further differences between illness samples were identified using (*post hoc*) Tukey's HSD test. Multivariate analysis of variance (MANOVA) was not used for analysis of differences in measures due to the moderate level of intercorrelation between *Specific-Concerns* and *General-Harm* ($\rho = 0.31$; $n = 524$; $p < 0.01$) and *General-Overuse* ($\rho = 0.24$; $n = 524$; $p < 0.01$). Differences in mean BMQ-General scores between Allopathic and Complementary care seekers was assessed using an independent samples *t*-test. A one-tailed test was used as the direction of association had been specified within the relevant hypothesis.

RESULTS

Reliability and Scale Intercorrelation

Cronbach alpha values obtained for each of the diagnostic group are shown in Table 11. These data indicate that both the BMQ-Specific and the BMQ-General scales have satisfactory internal consistency, with the exception of the *General-Harm* scale in three of the diagnostic groups. As both the psychiatric and diabetic samples had received all the items which subsequently comprised the Specific-Concerns scale Cronbach alpha values could be calculated for this scale. However, only 3 of the 5 *Specific-Necessity* items were included in the original item-pool administered to the diabetic sample and 4 of the 5 were included in the pool originally administered to the psychiatric sample. Therefore, for the psychiatric and diabetic samples, Cronbach alpha values were calculated for a 3 and 4-item *Specific-Necessity* scale respectively. A total of 31 of the asthmatic sample ($n = 78$)

Table 11 Internal consistency (Cronbach alpha) for the BMQ scales and test-retest correlations

	Asthmatic ($n = 78$)	*Diabetic* ($n = 99$)	*Renal* ($n = 47$)	*Cardiac* ($n = 116$)	*Psychiatric* ($n = 89$)	*General medical* ($n = 90$)	*Test-retest asthmatic patients* ($n = 31$)
Specific-Necessity	0.80	0.74[†]	0.55	0.76	0.74[‡]	0.86	0.77*
Specific-Concerns	0.75	0.80	0.73	0.76	0.63	0.65	0.76*
General-Overuse	0.74	0.80	0.77	0.74	0.73	0.60	0.60*
General-Harm	0.47	0.66	0.83	0.51	0.70	0.51	0.78*

*$p < 0.001$.
The diabetic and psychiatric out-patient samples completed shortened versions of the *Specific-Necessity* scale ([†]4 items; [‡]3 items).

Downloaded by [Dalhousie University] at 12:27 10 November 2014

returned the repeat questionnaires, giving a 40% response rate. The correlation coefficients shown in Table 11 indicate that the test-retest reliability of the scales is within accepted limits. Correlations between BMQ scales are shown in Table 12.

Criterion-related Validity

1. *Specific-Necessity*. Evidence for the criterion-related validity of the *Specific-Necessity* scale was provided by the negative correlation between scale scores and responses to the statement: *"I can cope without my medicines"* ($\rho = -0.44$; $n = 78$; $p < 0.001$) as expected. As predicted there were also positive correlations with scores on the IPQ *Timeline* ($\rho = 0.49$; $n = 77$; $p < 0.001$) and *Identity* ($\rho = 0.24$; $n = 76$; $p < 0.05$) scales which measure perceived duration and subjective symptomatology of the illness.
2. *Specific-Concerns*. Scores for the asthmatic group were positively correlated with the statement: *'I cannot always trust my medicines'* ($\rho = 0.33$; $n = 78$; $p < 0.005$), and *'I would like to change my present treatment'* ($\rho = 0.37$; $n = 78$; $p < 0.001$). The hypothesis that *Specific-Concerns* would be associated with a desire for more information about medicines was confirmed by the significant negative correlation with responses to the statement: *'I have been given enough information about my medicines'* ($\rho = -0.45$; $n = 78$; $p < 0.001$). As hypothesised, a significant positive correlation was obtained between *Specific-Concerns* and beliefs about personal sensitivity to the adverse effects of medication as assessed by the Sensitive-Soma scale administered to the General Medical and Cardiac samples ($\rho = 0.5$, $n = 211$, $p < 0.001$).
3. *General-Harm* and *General-Overuse*. Correlation between *General-Harm* scores and responses to the single item statement *"It is better to do without medicines"* was as expected ($\rho = 0.23$; $n = 78$; $p < 0.05$). Responses to the statement *"I can cope without my medicines"* correlated significantly, in the predicted direction, with both the *General-Harm* ($\rho = 0.24$; $n = 77$; $p < 0.05$) and *General-Overuse* scales ($\rho = 0.34$; $n = 78$; $p < 0.005$). Correlations between the *General-Harm* and *Sensitive-Soma* scales ($\rho = 0.25$, $n = 91$, $p < 0.05$), although small in magnitude, were in the predicted direction and statistically significant.
4. *Adherence to treatment*. Correlations between BMQ scales and reported adherence assessed by the RAM scale to medication were as expected. *Specific Necessity* beliefs correlated with higher reported adherence ($\rho = 0.19$; $n = 210$, $p < 0.01$). Correlations between the RAM scale and the Specific-Concerns ($\rho = -0.28$; $n = 210$; $p < 0.001$), *General-Overuse* ($\rho = -0.19$; $n = 210$; $p < 0.01$) and *General-Harm* ($\rho = -0.06$; $n = 210$; $p > 0.05$) scales were all in the predicted direction, although those between the RAM and *General-Harm* scales failed to reach statistical significance.

Discriminant Validity

1. *BMQ-Specific scales*. Table 13 shows the results of a series of one-way analyses of variance (ANOVA), with (*a priori*) linear contrasts and (*post-hoc*) Tukey's HSD tests

Table 12 Correlation between BMQ scales (total $n = 524$)

	Specific-Necessity	*Specific-Concerns*	*General-Harm*
Specific-Concerns	−0.01		
General-Harm	−0.05	0.31*	
General-Overuse	−0.17	0.24*	0.40*

Note: *$p < 0.001$.

Downloaded by [Dalhousie University] at 12:27 10 November 2014

Table 13 Scale means and standard deviations for BMQ scales for the six illness groups comprising the main sample

Scale	*Asthmatic* $n=78$	*Diabetic* $n=99$	*Renal* $n=47$	*Cardiac* $n=116$	*Psychiatric* $n=85$	*General medical* $n=86$	*F* $df=5{,}505$	*P*
Specific-Necessity								
Mean	19.67_b	21.26_a	$19.45_{b,c}$	$18.72_{b,c}$	17.72_c	19.65_b	11.73	<0.01
SD	3.23	2.98	2.78	3.02	3.75	3.92		
Specific-Concerns								
Mean	15.76_a	12.91_c	13.77_c	13.95_c	$15.60_{a,b}$	14.26_b	7.49	<0.01
SD	4.09	3.38	4.28	3.73	3.36	3.92		
General-Harm								
Mean	10.24_a	9.29_a	9.91_a	9.98_a	9.92_a	9.86_a	1.29	0.26
SD	2.30	2.43	3.76	2.32	2.81	2.80		
General-Overuse								
Mean	$11.64_{a,b}$	11.43_a	$12.66_{a,b}$	12.80_b	$12.25_{a,b}$	$12.42_{a,b}$	3.48	0.01
SD	2.59	2.77	3.19	2.90	2.84	2.76		

Note: Means sharing a common subscript are not significantly different by (*a priori*) linear contrasts or (*post hoc*) Tukey's HSD test ($p > 0.05$).

Table 14 Group differences in BMQ-General scores for matched samples of orthodox and complementary patients

Measure		*Allopathic* ($n=36$)	*Complementary* ($n=36$)	*t* ($df=70$)	*P* (1-*tailed*)
General-Overuse	Mean	12.44	16.56	5.89	<0.001
	SD	3.26	2.62		
General-Harm	Mean	10.75	11.85	1.94	<0.05
	SD	2.61	2.20		

in which mean scores on the BMQ scales were compared across illness samples. It can be seen that the BMQ scales were able to distinguish between patients on the basis of illness (and treatment) groupings. The predictions for discriminant validity of the *Specific-Necessity* scale were confirmed by the finding that diabetic group had significantly higher *Specific-Necessity* scores than all other groups and the asthmatic patients had significantly higher scores than the psychiatric outpatients who attained the lowest mean as predicted. As was expected, the asthmatic and psychiatric samples had significantly higher *Specific-Concerns* than the other illness groups, supporting the discriminant validity of this scale.

2. *BMQ-General scales*. As was predicted, patients attending a Complementary clinic (homeopath/herbalist) had significantly higher scores on both the *General-Overuse* and *General-Harm* scales than those presenting a personal prescription for dispensing at a community pharmacy, as shown in Table 14.

Downloaded by [Dalhousie University] at 12:27 10 November 2014

Downloaded by [Dalhousie University] at 12:27 10 November 2014

DISCUSSION

Exploratory PCA of commonly-held beliefs about medication prescribed for a specific illness (Specific beliefs) and more general beliefs about medicines as a whole (General beliefs) produced simple factor structures which were subsequently verified by confirmatory factor analysis. Replication of factor structures in different illness samples showed an acceptable degree of stability and suggested that the factors represent 'core themes' underpinning common representations of Specific and General medication.

The core themes relating to medication prescribed for the patient were: beliefs about the necessity of the medicines for maintaining health (*Specific-Necessity*) and concerns about medication (*Specific-Concerns*). The *Specific-Necessity* construct represents the perceived role of medication in protecting against deterioration of the present and future health status of the patient. The *Specific-Concerns* construct comprises aspects of both an emotional (e.g. "*Having to take my medicines worries me*") and a cognitive ("*My medicines are a mystery to me*") representation and thus may provide access to both aspects of the parallel processing described by Leventhal in the SRM (Leventhal *et al.*, 1980)

Both the general factors contain items relating to aspects of medication which are essentially negative and a coherent "benefit" dimension did not emerge from our original items. This may be because the items we used were not representative of an underlying dimension of "benefit". Alternatively, it may simply be that a clear representation of benefit is obscured by strong beliefs about the potential for harm. It is salient that in most of the studies from which the item pool was derived, the benefit of medicines was often taken for granted. People who had generally negative views about medication tended to cite the potential for harm, rather than the lack of "efficacy" or "benefit" as a focus for their concerns about medication (Conrad, 1985; Morgan and Watkins, 1988) and other authors have remarked on this (Fallsberg, 1991). At first sight, the representations of medicines in general encompassed by the BMQ-General scales seem to amount to a rather negative view of medicines as harmful and overused by doctors. However, this does not necessarily mean that most people see medicines in this way. It is possible to disagree with the statements on each factor and so express a view of medication as essentially safe and appropriately used. The main point here is that PCA showed that certain medication beliefs (e.g. about addiction, poison, harm, regular long term use) could be organised into coherent themes relating to the nature of medicines (*General-Harm*) and views about how they are used by doctors (*General-Overuse*).

Measures of internal consistency and test-retest reliability of the BMQ scales were encouraging as was the criterion-related and discriminant validity data. Expected correlations were obtained between BMQ scale scores and other measures of illness and medication beliefs and between *Specific-Concerns* and self-reported adherence to medication. The BMQ scales were able to distinguish between different illness groups/treatment modalities, between particular adherence behaviours and between users of allopathic and complementary therapies.

The internal consistency of the *General-Harm* sub-scale was disappointing in three data sets (asthmatic, cardiac and general medical). Examination of Cronbach alpha values following individual item deletions showed that this could not be attributed to a single "rogue item" but was a true reflection of low internal consistency. However, in other data sets this scale had a greater degree of internal consistency. The reason for this disparity is unclear but seems to support the premise that patients with certain illnesses tend to develop a more coherent representation of medication in general, which is perhaps influenced by their

personal experience with prescribed medication. We are currently conducting further studies on the cognitive representation of beliefs about medicines in general using other samples in an attempt to resolve these issues. In the meantime we recommend that the General-Harm scale is used with caution.

The scope of the present evaluation is limited by the fact that, due to the lack of availability of validated measures of medication beliefs, aspects of the criterion-related validity of the BMQ scales were evaluated against single-item constructs of attitudes to specific and general medication. The evaluation of the validity of the BMQ was also limited by the absence of data testing the predictive validity of the measure. This is currently being evaluated by examining inter-relations between BMQ scales and other variables separated over a 3-month period. Despite these limitations the data described above provide preliminary evidence for the criterion-related validity, discriminant validity and the reliability of the BMQ scales and support its use as a research tool within the context of studies investigating peoples' beliefs about medication.

The BMQ-Specific is a flexible instrument which can be adapted to assess beliefs about all medicines for a particular condition or for individual components of the regimen. This can be achieved by changing the reference statement associated with the questionnaire as shown in the Appendix. We have also developed versions to assess partner or carer's views about a patient's medication, and parents' perceptions of medication prescribed for their child. (Partner and parent versions are available on request from the authors). The Specific and General questionnaires may be used separately or in combination.

In conclusion, the data presented in this paper confirm the value of the BMQ as a novel method for assessing beliefs which patients commonly hold about their prescribed medication and about medicines in general. We hope that the measure will facilitate further research into patients' perspectives of treatment.

Downloaded by [Dalhousie University] at 12:27 10 November 2014

Acknowledgements

This research was supported by a research award from the Pharmacy Enterprise Scheme, Department of Health, UK. We would also like to acknowledge a number of colleagues who made important contributions to the development of this questionnaire. Early discussions with Professor Marie Johnston helped to focus our ideas. Thanks are due to Professor Howard Leventhal and colleagues for permission to use the Sensitive Soma scale and to Associate Professor Keith Petrie for helpful discussions. Railton Scott, Angela Lashau, Barry Jubraj and Carol Kirkman helped with data collection from the asthmatic, diabetic, renal and psychiatric illness groups. Thanks are also due to Alice Ward, Linda Dodds and the pre-registration pharmacists of South East Thames who collected data from the cardiac and general medical groups. We are also grateful to Professor Richard Vincent and Drs Michael Rosenberg, John Hartley, Charles Turton (Brighton) and to Dr David Taube and Sian Sumner (London) for their help and to those clinicians who provided access to their patients and to the patients who took part. We would also like to thank the two anonymous reviewers for their helpful comments.

References

Abraham, C., Sheeran, P., Spears, R. and Abrams, D. (1992) Health beliefs and the promotion of HIV-preventive intentions among teenagers: a Scottish perspective. *Health Psychology* **11**, 363–370.

Abraham, C. and Hampson, S.E. (1996) A social cognition approach to health psychology: philosophical and methodological issues. *Psychology and Health* **11**, 223–241.

Ajzen, I. (1985) From intentions to actions: a theory of planned behaviour. In: *Action-Control: From Cognition to Behaviour*, ed. Kuhl, J. and Beckmann, J. Heidelberg: Springer-Verlag.

Ajzen, I. and Fishbein, M. (1980) *Understanding attitudes and predicting social behaviour*, Englewood Cliffs, NJ: Prentice-Hall.

Becker, M.H., Radius, S.M., Rosenstock, I.M., Drachman, R.H., Schubert, K.C. and Teets, K.C. (1978) Compliance with a medical regimen for asthma: a test of the health belief model. *Public Health Reports* **93**, 268–277.

Britten, N. (1994) Patients' ideas about medicines: a qualitative study in a general practice population. *British Journal of General Practice* **44**, 465–468.

Brownlee-Duffeck, M., Peterson, L., Simonds, J.F., Goldstein, D., Kilo, C. and Hoette, S. (1987) The role of health beliefs in the regimen adherence and metabolic control of adolescents and adults with diabetes mellitus. *Journal of Consulting and Clinical Psychology* **55**, 139–144.

Cameron, L., Leventhal, E.A. and Leventhal, H. (1993) Symptom representations and affect as determinants of care seeking in a community-dwelling, adult sample population. *Health Psychology* **12**, 171–179.

Cattell, R.B. (1995) The fallacy of five factors in the personality sphere. *The Psychologist* **8**, 207–208.

Conner, M. and Norman, P. (1996) *Predicting health behaviour: research and practice with social cognition models*, Buckingham: Open University Press.

Cochran, S.D. and Gitlin, M.J. (1988) Attitudinal correlates of Lithium compliance in bipolar affective disorders. *The Journal of Nervous and Mental Disease* **176**(8): 457–464.

Conrad, P. (1985) The meaning of medications: another look at compliance. *Social Science in Medicine* **20**(1): 29–37.

Cummings, K.M., Becker, M.H., Kirscht, J.P. and Levin, N.W. (1981) Intervention strategies to improve compliance with medical regimens by ambulatory haemodialysis patients. *Journal of Behavioural Medicine* **4**, 111–127.

Diefenbach, M., Leventhal, H. and Leventhal, E.A. (1996). The Sensitive Soma Scale. Manuscript in preparation.

DiMatteo, M.R., Sherbourne, C.D., Hays, R.D., Ordway, L., Kravitz, R.L., McGlynn, E.A., Kaplan, S. and Rogers, W.H. (1993) Physicians' characteristics influence patients' adherence to medical treatment: results from the Medical Outcomes Study. *Health Psychology* **12**, 93–102.

Donovan, J.L. and Blake, D.R. (1992) Patient non-compliance: Deviance or reasoned decision-making? *Social Science in Medicine* **34**, 507–513.

Echabe, A.E., Guillen, C.S. and Ozamiz, J.A. (1992) Representations of health illness and medicines: coping strategies and health promoting behaviour. *British Journal of Clinical Psychology* **31**, 339–349.

Fallsberg, M. (1991) *Reflections on medicines and medication: a qualitative analysis among people on long-term drug regimens*, Linköping, Sweden: Linkoping University.

Horne, R. (1993) One to be taken as directed: reflections on non-adherence (non-compliance). *Journal of Social and Administrative Pharmacy* **10**, 150–156.

Horne, R. (1997) Representations of medication and treatment: advances in theory and measurement. In: *Perceptions of Health and Illness: Current Research and Applications*, ed. Petrie, K.J. and Weinman, J. London: Harwood Academic, pp. 155–187.

Horne, R. and Weinman, J. (1998) Predicting treatment adherence: an overview of theoretical models. In: *Adherence to Treatment in Medical Conditions*, ed. Myers, L. and Midence, K. London: Harwood Academic pp. 25–50.

Horwitz, R.I., Viscoli, C.M., Berkman, L., Murray, C.J., Ransohoff, D.F. and Sindelar, J. (1990) Treatment adherence and the risk of death after a myocardial infarction. *Lancet* **336**, 542–545.

Horwitz, R.I. and Horwitz, S.M. (1993) Adherence to treatment and health outcomes. *Archives of Internal Medicine* **153**, 1863–1868.

Janz, N.K. and Becker, M.H. (1984) The health belief model: a decade later. *Health Education Quarterly* **11**, 1–47.

Kelly, G.R., Mamon, J.A. and Scott, J.E. (1987) Utility of the health belief model in examining medication compliance among psychiatric outpatients. *Social Science and Medicine* **25**, 1205–1211.

Kline, P. (1994) *An Easy Guide to Factor Analysis*, London: Routledge.

Kravitz, R.L., Hays, R.D., Sherbourne, C.D., DiMatteo, M.R., Rogers, W.H., Ordway, L. and Greenfield, S. (1993) Recall of recommendations and adherence to advice among patients with chronic medical conditions. *Archives of Internal Medicine* **153**, 1869–1878.

Leventhal, H., Meyer, D. and Nerenz, D. (1980) The common sense representation of illness danger. In S. Rachman (Ed.) *Contributions to Medical Psychology*. Oxford: Pergamon Press.

Leventhal, H. and Cameron, L. (1987) Behavioural theories and the problem of compliance. *Patient Education and Counselling* **10**, 117–138.

Leventhal, H., Easterling, D.V., Coons, H.L., Luchterhand, C.M. and Love, R.R. (1986) Adaptation to chemotherapy treatments. In: Andersen, B.L. (Ed.) *Women with Cancer: Psychological Perspectives*, pp. 172–203. New York: Springer Verlag.

Downloaded by [Dalhousie University] at 12:27 10 November 2014

Leventhal, H. and Diefenback, M. (1991) The Active Side of Illness Cognition. In: Skelton, J.A. and Croyle, R.T. (Eds.) *Mental Representation in Health and Illness*, pp. 245–271. New York: Springer Verlag.
Leventhal, H., Diefenbach, M. and Leventhal, E.A. (1992) Illness cognition: using common sense to understand treatment adherence and affect cognition interactions. *Cognitive Therapy and Research* **16**(2), 143–163.
Leventhal, H., Benyamini, Y., Brownlee, S., Diefenbach, D., Leventhal, E.A., Patrick-Miller, L. and Robitaille, C. (1997) Illness representations: theoretical foundations. In: *Perceptions of Health and Illness: Current Research and Applications*, ed. Petrie, K.J. and Weinman, J. London: Harwood Academic, pp. 19–46.
Lorish, C.D., Richards, B. and Brown, S. (1990) Perspective of the patient with rheumatoid arthritis on issues related to missed medication. *Arthritis Care and Research* **3**, 78–84.
Marinker, M. (1997) From compliance to concordance: achieving shared goals in medicine taking. *British Medical Journal* **314**, 747–748.
Marteau, T.M. (1990) Attitudes to doctors and medicine: the preliminary development of a new scale. *Psychology and Health*, **4**, 351–356.
Marteau, T.M. (1995) Health Beliefs and attributions. In: Broome, A. and Llewellyn, S. (Eds.) *Health Psychology: Processes and Applications*, 2nd edn. pp. 3–20. London: Chapman and Hall.
Meyer, D. and Leventhal, H. (1984) Common sense models of illness: the example of hypertension. *Health Psychology* **4**(2), 115–135.
Meichenbaum, D. and Turk, D.C. (1987) *Facilitating treatment adherence: a practitioner's handbook*, New York: Plenum Press.
Morgan, M. and Watkins, C.J. (1988) Managing hypertension: beliefs and responses to medication among cultural groups. *Sociology of Health and Illness* **10**, 561–578.
Morisky, E.E., Green, L.W. and Levine, D.M. (1986) Concurrent and predictive validity of a self-reported measure of medication adherence. *Medical Care* **24**, 67–74.
Moss-Morris, R., Petrie, K.J. and Weinman, J. (1996) Functioning in Chronic Fatigue Syndrome: do illness perceptions play a regulatory role? *British Journal of Health Psychology* **1**, 15–25.
Norusis, M.J. (1992) *SPSS for Windows: Professional Statistics (Release 5)*, Chicago: SPSS Inc.
Petrie, K.J., Weinman, J., Sharpe, N. and Buckley, J. (1996) Predicting return to work and functioning following myocardial infarction: the role of the patient's view of their illness. *British Medical Journal* **312**, 1191–1194.
Redeimeier, D.A., Rozin, P. and Kahneman, D. (1993) Understanding patients' decisions: cognitive and emotional perspectives. *Journal of the American Medical Association* **270** (1), 72–76.
Ried, L.D. and Christensen, D.B. (1988) A psychosocial perspective in the explanation of patients' drug-taking behaviour. *Social Science in Medicine* **27**, 277–285.
Ried, L.D., Oleen, M.A., Martinson, O.B. and Pluhar, R. (1985) Explaining intention to comply with antihypertensive regimens: the utility of health beliefs and the theory of reasoned action. *Journal of Social and Admistrative Pharmacy* **3**(2): 42–52.
Rosenstock, I. (1974) The health belief model and preventative health behaviour. *Health Education Monographs* **2**, 354–386.
Royal Pharmaceutical Society of Great Britain (1997) *From compliance to concordance: achieving shared goals in medicine taking*. London.
Skelton, J.A. and Croyle, R.T. (1991) *Mental Representation in Health and Illness*, New York: Springer-Verlag.
Streiner, D.L. (1994) Figuring out factors: the use and misuse of factor analysis. *Canadian Journal of Psychiatry* **39**(3): 135–140.
Tabachnik, B.G. and Fidell, L.S. (1989) *Using multivariate statistics*, 2nd edn. New York: Harper Collins.
Weinman, J., Petrie, K.J., Moss-Morris, R. and Horne, R. (1996) The Illness Perception Questionnaire: a new method for assessing cognitive representations of illness. *Psychology and Health* **11**, 431–445.
Woller, W., Kruse, J., Winter, P. and Mans, E.J. (1993) Cortisone image and emotional support by key figures in patients with bronchial asthma: an empirical study. *Psychotherapy and Psychosomatics* **59**, 190–196.

APPENDIX: BMQ ITEMS

BMQ-Specific

*Your views about medicines prescribed for you**

- We would like to ask you about your personal views about medicines prescribed for you.
- These are statements other people have made about their medicines.
- Please indicate the extent to which you agree or disagree with them by ticking the appropriate box.
- There are no right or wrong answers. We are interested in your personal views.

Downloaded by [Dalhousie University] at 12:27 10 November 2014

Rated: strongly agree, agree, uncertain, disagree, strongly disagree

My health, at present, depends on my medicines
Having to take medicines worries me
My life would be impossible without my medicines
Without my medicines I would be very ill
I sometimes worry about long-term effects of my medicines
My medicines are a mystery to me
My health in the future will depends on my medicines
My medicines disrupt my life
I sometimes worry about becoming too dependent on my medicines
My medicines protect me from becoming worse

Note:
To elicit beliefs about individual components of the treatment regimen the reference statement should refer to the medicine by name e.g. *Your views about aspirin prescribed for you* Additionally items can refer to a named illness e.g. *Your views about medicines prescribed for your asthma*

BMQ-General

Your views about medicines in general

- We would like to ask you about your personal views about medicines in general.
- These are statements other people have made about medicines in general.
- Please indicate the extent to which you agree or disagree with them by ticking the appropriate box.
- There are no right or wrong answers. We are intersted in your personal views.

Rated: strongly agree, agree, uncertain, disagree, strongly disagree

Doctors use too many medicines
People who take medicines should stop their treatment for a while every now and again
Most medicines are addictive
Natural remedies are safer than medicines
Medicines do more harm than good
All medicines are poisons
Doctors place too much trust on medicines
If doctors had more time with patients they would prescribe fewer medicines.

Downloaded by [Dalhousie University] at 12:27 10 November 2014
