# Supplementary material for: Awareness and practical evaluation of correct use of iron chelators; a study to track the ambiguities of thalassemia patients on their medications in Iran
Source: BMC Res Notes. 2024 Jun 13;17:163. doi: 10.1186/s13104-024-06819-3 (PMC11170868; doi:10.1186/s13104-024-06819-3)
Supplement: Supplementary file 2 — Supplementary Material 2 [file 13104_2024_6819_MOESM2_ESM.pdf]

2022

Journal of Pharmaceutical Care

jpc.tums.ac.ir

Development of Thalassemia Medication Questionnaire (TMQ): An Instrument for Measuring Major Thalassemia Patients' Knowledge and Practice Regarding their Medications

Azita Azarkeivan[1], Ghader Mohammadnezhad[2], Hadi Esmaily[3*]

[1]Blood Transfusion Research Center, High Institute for Research and Education in Transfusion Medicine, Thalassemia Clinic, Tehran, Iran.

[2]School of Pharmacy, Shahid Beheshti University of Medical Sciences, Tehran, Iran.

[3]Department of Clinical Pharmacy, School of Pharmacy, Shahid Beheshti University of Medical Sciences, Tehran, Iran.

Received: 2022-10-19, Revised: 2022-11-21, Accepted: 2022-11-23, Published: 2022-12-31

ARTICLE INFO

Article type:
Original article

Keywords:
Iron Chelating Agents;
Deferasirox;
Deferoxamine;
Deferiprone;
Thalassemia;
Questionnaire

ABSTRACT

Background: Thalassemia is a congenital disease and Iran is as one of the countries in the thalassemia belt. It has a huge burden in Iranian national health budget. The aim of this study was to develop a questionnaire to assess the knowledge and practice of thalassemia patients toward their medications.

Methods: This is a methodological research, which was done from April 2020 to November 2021 in Tehran, Iran, To develop a valid and reliable instrument for measuring major thalassemia patients' knowledge and practice regarding their medications, a questionnaire based on three iron chelating medications (deferoxamine, deferiprone, and deferasirox) developed. This process done by holding several expert panel meetings. This questionnaire was consisting of some aspects such as: administration, self-monitoring, miss dose, common interactions, and adverse events. Content validity index (CVI) and internal consistency and reliability were calculated.

Results: The CVI was as 0.76 for deferoxamine, 0.80 deferiprone and 0.88 for deferasirox questionnaire. Further analysis for internal consistency demonstrated satisfactory results with Cronbach's alpha coefficients ranging from 0.743 to 0.781. Results showed Thalassemia Medication Questionnaire (TMQ) demonstrated acceptable validity and reliability for application.

Conclusion: It was concluded that TMQ could be a useful instrument to measure knowledge and practice about iron chelation therapy. The reason is the diverse range of questions and the simplicity, validity, reliability, and the practicability.

J Pharm Care 2022; 10(4): 223-228.

► **Please cite this paper as:**
Azarkeivan A, Mohammadnezhad Gh, Esmaily H. Development of Thalassemia Medication Questionnaire (TMQ): An Instrument for Measuring Major Thalassemia Patients' Knowledge and Practice Regarding their Medications. J Pharm Care 2022; 10(4): 223-228.

Introduction

Thalassemia is a congenital hemoglobinopathy that can be life-threatening if not properly managed. Iran is located in a high-prevalence belt with about 5-10% of beta-thalassemia traits; however, its prevalence has declined in the last decades due to screening programs for beta-thalassemia traits before marriage (1). Increasing the iron levels in plasma is the most important problem in patients with major thalassemia. An iron overload could be controlled with a continuous chelating agent capable of complexion with iron and promoting its excretion. Deferoxamine (DFX) has for decades been the standard for iron chelation therapy (ICT) worldwide (2). In addition, currently, there are two oral iron chelators in Iran: Deferiprone (DFP) and Deferasirox (DFS); DFP is an effective oral iron chelator able to reduce iron overload and maintain a safe body iron level (3). However, serious adverse effects (AEs) associated with DFP include neutropenia, agranulocytosis, and routine

**Corresponding Author: Dr Hadi Esmaily*
Address: Department of Clinical Pharmacy, School of Pharmacy, Shahid Beheshti University of Medical Sciences, Tehran, Iran.
Email: Esmaily_hadi@sbmu.ac.ir

Copyright © 2022 Tehran University of Medical Sciences.
This work is licensed under creative Commons Attribution-NonCommercial 4.0 International license (https://creativecommons.org/licenses/by-nc/4.0/). Noncommercial uses of the work are permitted, provided the original work is properly cited

monitoring at frequent regular intervals is recommended (4). DFS is also simple to use, with a once-daily dosing schedule, and it has a manageable safety profile and is well-accepted by the patients, which is likely to improve compliance (5). Although ICT is the most recommended solution in thalassemia treatment, most patients do not have acceptable adherence to ICT. There are many obstacles to the successful outcomes of these medications, such as unpleasant injections, life-long need, many AEs, and lack of patient knowledge about the misuse of medications and the dangers of increased iron levels (6). It is essential to develop an instrument to measure the patients' knowledge and practice of their medications because one of the most practicable and affordable ways to improve treatment outcomes is to improve the knowledge and practice of patients about their medications. It is critical for thalassemia patients to know the essential points about their medications, AEs, monitoring parameters, and interactions. These two factors lead to an acceptable adherence that improves the treatment outcomes and decreases their disease burden (7,8).

To the best of our knowledge, there is no instrument for measuring thalassemia patients' knowledge and practice about their medications. The present research is novel in this field, and due to the lack of suitable instruments in this field, the present study was designed as a necessity with the aim of developing and psychometric evaluation of the "Thalassemia Medication Questionnaire" (TMQ).

Methods

This is methodological research done from April 2020 to November 2021 in Tehran, Iran. In the first step, we prepared the primary version of the knowledge and practice questionnaire for thalassemia based on the data obtained from the textbooks, articles, and online monographs of UpToDate® in English (9). We extracted the critical patient educational points that thalassemia patients should know. Also, several review articles about ICTs were studied to pick up all relevant points (10-15). The similar points which represent the same content merged together. Then the primary version of the questionnaire was designed. The questions of knowledge were divided into three categories based on the three mentioned agents as ICTs, and questions for the practice part of the questionnaire. Then appearance, content, and structure validity of the questionnaire were assessed.

In the second step, two fields of faculty members were involved in evaluating the appearance and validity of the questionnaire. This panel consists of seven experts in the field of clinical pharmacy, public health, and hematology. This panel held several meetings, and after receiving the opinions of these professors, the necessary changes to the questions were made, including (font, pagination, section arrangement, etc.). After reviewing the professors' opinions, the questionnaire was approved for appearance validity.

In the third step, to evaluate the content validity quantitatively, the content validity ratio (CVR) and content validity index (CVI) method of Waltz & Bausell were used (16). The content validity of the patient's knowledge questionnaire was assessed according to CVI by ten professors other than experts who were involved in the primary step. The aforementioned experts had extensive expertise in the research field and had done some similar studies. Furthermore, we requested the respondents to suggest deleting any irrelevant items or making any necessary changes to the items to improve transparency everywhere needed. For assessing the CVI, at first, the relevance of each statement in the questionnaire was evaluated based on a three-part indicator with four grades (one to four). If the score for each question was equal to or greater than 79%, the phrase in the questionnaire was maintained. If the score was between 70-79%, the phrase was modified and revised; and if it was less than 70%, the phrase was deleted (17). Based on this index, the clarity and simplicity of phrases were also examined. In order to measure the CVR, we used Lawshe Table. For this reason, ten faculty members from different universities of the country and experts in the field of hematology who had expertise in the treatment of thalassemia major were asked to determine the necessity of each statement in a 3 part of Likert's spectrum: "It is necessary" = 3, "It is useful but not necessary" = 2, "It is not necessary" = 1. Then, based on the following formula, the CVR of the questionnaires was calculated (18). Finally, 17 questions for DFX, ten for DFP, and ten for DFS were selected, and the practice part of the questionnaire included six questions.

In the fourth step, we used Cronbach's alpha method to determine the reliability of the knowledge questionnaire in patients with major thalassemia. For this reason, 87 patients were involved in three different groups based on three ICT. The reliability of the questionnaire was assessed by completing it with 30 target population. By obtaining Cronbach's alpha coefficient, we were able to determine the reliability of the questionnaire, and it turned out that this tool would give the same results in the same conditions.

The fifth step, the Persian translation of the questionnaire, was carried out in a forward-backward translation procedure. The original Persian questionnaire was translated into English by two bilingual and native Farsi-speaking translators; then reverse translation was done. This English version was translated into Farsi by another expert who did not see the original questionnaire, and then the re-translated questionnaire was turned into English language and was compared to the original questionnaire for validation of translation. Statistical Package for the Social Sciences (SPSS, IBM Corp., Armonk, NY, USA) software version 25 was used for statistical analysis.

Results

All of the ten involved experts at this stage completed the content validity forms. Using the ratings and comments provided by content experts, we performed the following analysis to assess the content validity of the developed instrument.

To assess the relevance of the questionnaire, we evaluated the item content validity index for the relevance of each of the questions in the developed instrument. In the 16 questions for DFX, eight questions for DFP, and seven questions for DFS, this index was greater than the acceptable level of 78%. After reviewing the content validity, 1 question of the questions of DFX, two from the DFP questions, and three of the DFS questions was deleted, and validity was re-calculated. The values of the content validity index were showed in Table 1.

Table 1. Item's content validity ratio and content validity index for relevance, clarity, and simplicity for deferoxamine, deferiprone, deferasirox.

Knowledge Questions for DFX	CVR	CVI Clarity	CVI Relevance	CVI Simplicity
1- Which location is the best injection site to administer DFX?	0.80	0.70	1.00	1.00
2- Which option is the best method for injecting DFX?	1.00	0.70	1.00	1.00
3- What is the best way to reduce pain when DFX is injected?	1.00	0.90	0.90	1.00
4- At least how much distilled water is required to dilute each vial of 500 mg DFX?	1.00	0.70	0.90	0.90
5- Which vitamin should be used after the first month of treatment?	0.40	0.90	1.00	1.00
6- Which item is correct about how to inject DFX?	0.60	0.60	1.00	0.90
7- Which IV fluid is compatible with DFX, and can be injected simultaneously?	0.80	0.80	0.90	1.00
8- Which option is correct about using the vitamin C?	0.40	0.80	0.60	0.90
9- Which item is the storing condition of DFX vials?	0.80	0.90	1.00	0.90
10- How long DFX can be used after reconstitution?	1.00	1.00	1.00	0.80
11- How often you should do the blood tests during treatment?	0.60	0.90	0.90	0.70
12- What is the best way to inject DFX with blood simultaneously?	0.40	1.00	1.00	1.00
13- What kind of physical examinations should be taken at regular intervals while taken DFX?	0.80	0.80	0.90	0.90
14- What is the most common side effect of DFX?	1.00	0.80	1.00	1.00
15- Which item is indicative of the sensitivity reaction to DFX and if I noticed that, DFX must be stopped until consulting with my doctor?	1.00	0.90	1.00	1.00
16- What approach should be taken if a dose is missed?	0.60	0.90	1.00	1.00
**Knowledge Questions for DFP**				
?What kind of laboratory and medical tests should be taken on a regular basis while receiving DFP 1-	0.60	0.90	1.00	1.00
2- What is the first step to reduce the DFP-induced nausea?	0.80	0.70	0.80	0.80
3- How often blood test is needed while taking DFP?	1.00	0.90	0.90	0.90
4- Which element should be monitored closely in the blood tests while taking DFP?	1.00	1.00	1.00	1.00
5- If you noticed which reaction you should call the doctor right away?	0.60	0.80	1.00	1.00
6- Which supplement should not be taken while receiving DFP?	0.80	0.90	1.00	1.00
7- When I take DFP, what should I pay the most attention to?	0.80	0.90	1.00	1.00
8- What approach should be taken if a dose is missed?	0.80	0.80	1.00	1.00
**Knowledge Questions for DFS**				
1- What kind of medical examinations and laboratory tests should be taken on a regular basis while receiving DFS?	0.60	0.80	0.90	0.90
2- What is the best way to take DFS?	1.00	0.90	1.00	1.00
3- When is the best time to take DFS?	1.00	0.90	1.00	1.00
4- Which fluid can be used instead of water to take DFS?	1.00	1.00	1.00	1.00
5- Which of the following medicines cannot be taken with DFS?	0.80	1.00	1.00	1.00
6- What approach should be taken if a dose is missed?	1.00	0.80	1.00	0.90
7- What is the storage condition for DFS?	0.80	0.40	1.00	1.00

*DFX: Deferoxamine, DFP: Deferiprone, DFS: Deferasirox, IV: intravenous.

According to the number of professors and experts in this field (ten people), the minimum CVR should be 0.69. Therefore, the questions' content validity were 0.76, 0.80, and 0.89 respectively for DFX, DFP, and DFS which were accepted. Questions have been given to each patient, depending on the type of ICT used.

For reliability assessment, The Cronbach's alpha coefficient test was assessed for each questionnaire in 87 patients, which confirms acceptable internal consistency of the instrument. Cronbach's alpha of each question was shown in details in Table 2. Demographic characteristics of participants are presented in Table 3.

Table 2. Reliability Statistics.

DFP		DFS		DFX			
Items	Cronbach's Alpha if Item Deleted	Items	Cronbach's Alpha if Item Deleted	Items	Cronbach's Alpha if Item Deleted	Items (*Con*)	Cronbach's Alpha if Item Deleted
Q1	0.743	Q1	0.714	Q1	0.889	Q9	0.889
Q2	0.699	Q2	0.618	Q2	0.881	Q10	0.881
Q3	0.771	Q3	0.713	Q3	0.894	Q11	0.894
Q4	0.743	Q4	0.714	Q4	0.889	Q12	0.889
Q5	0.699	Q5	0.618	Q5	0.881	Q13	0.881
Q6	0.800	Q6	0.745	Q6	0.900	Q14	0.900
Q7	0.817	Q7	0.789	Q7	0.906	Q15	0.906
Q8	0.743			Q8	0.889	Q16	0.889
**Cronbach's Alpha of 8 Items**	0.781	**Cronbach's Alpha of 7 Items**	0.743	**Cronbach's Alpha of 16 Items**			0.743

*DFX: Deferoxamine, DFP: Deferiprone, DFS: Deferasirox, Q: question.

Table 3. Demographic characteristics of the participants.

	DFX	DFP	DFS
**Number**	30	31	26
**Age:** range (Mean ± SD)	16-40 (28.30 ± 6.49)	19-45 (31.74 ± 6.13)	19-47 (29.88 ± 6.86)
**Sex**			
**Female** (%)	13 (43.3%)	13 (30.2%)	8 (30.8%)
**Male** (%)	17 (56.7%)	18 (41.9%)	18 (69.2%)
**Education**			
**Under Diploma** (%)	3 (10%)	2 (4.7%)	3 (11.5%)
**Diploma** (%)	11 (36.7%)	11 (25.6%)	8 (30.8%)
**Academic Degree** (%)	16 (53.3%)	18 (41.9%)	15 (57.7%)
**Marital Status**			
Single (%)	22 (73.3%)	22 (71.0%)	22 (84.6%)
Married (%)	8 (26.7%)	9 (20.9%)	4 (15.4%)

*DFX: Deferoxamine, DFP: Deferiprone, DFS: Deferasirox, SD: standard deviation.

The practice part of the questionnaire is presented in the Table 4. The answers of the thalassemia patients' practice questions were evaluated using the model key answer sheet, which was prepared by the expert panel and graded as acceptable practice; scores more than 75%, average practice; 50–75% and poor practice scores less than 50%.

Table 4. Practice content validity ratio and content validity index for relevance, clarity, and simplicity.

Practice Questionnaire	CVR	CVI Clarity	CVI Relevance	CVI Simplicity
1- Does the patient take the medicine at the correct way?	1.00	0.90	1.00	1.00
2- Does the patient take the medicine at the correct time?	1.00	1.00	1.00	1.00
3- Does the patient have an accurate approach to the missed dose?	0.90	1.00	1.00	1.00
4- Does the patient perform the right job in dealing with the adverse effects of the medicine?	1.00	1.00	1.00	1.00
5- Does the patient perform the right job to monitor the responses to treatment?	1.00	1.00	0.90	1.00
6- Does the patient store the medicine in a right way?	1.00	1.00	1.00	1.00

Discussion

In this study a valid and reliable questionnaire for the assessment of patients' knowledge, and practice towards thalassemia medications was developed in Persian and after validations it was translated to English. The questionnaire validity to measure patients' knowledge towards thalassemia is based on two different assessments. First, we applied a quantitative approach for content validity assessment. The content validity assessment uses expert's views for evaluating simplicity, relevance, and clarity of the new instrument for each of the items and the whole scale. This approach was applied for instrument's development in other fields of health services. To the best of our knowledge, this is the first investigation which developing an instrument to assess knowledge, and practice of thalassemia patients about their medications. Hence, this instrument may also act as a prototype for other researchers' eager to develop instruments for measuring patient's knowledge towards thalassemia in other languages. According to the Table 1 to 2, the scale content validity index for relevance and clarity of this instrument for each question was higher than 0.79 and questions with a lower index of this amount were removed. We evaluated the reliability of this instrument using Cronbach's alpha. The Cronbach' alpha was assessed to evaluate the internal consistency of the instrument. Results for the internal consistency of the instrument showed Cronbach's alpha of 0.73, which is greater than the acceptable level of 0.60. The nature of knowledge which is not repeatable may negatively affects the Cronbach's alpha. Hence, this Cronbach's alpha is expectable. Although values higher than 0.90 can suggest a high level of item redundancy, we decided to eliminate any question from the instrument if Cronbach's alpha continues with values higher than 0.90. Thus, the questionnaire benefited from acceptable face and content validity to assess knowledge among thalassemia patients, which is consistent with the results of other studies. In the other study, the content value and reliability of the health-promoting lifestyle of adolescents with beta thalassemia were calculated, using CVR, CVI and Cronbach's alpha methods, which were 0.92, 0.96, and 0.94, respectively (19).

According to the Table 4, the practice of patients towards their medications was measured. It is suggested that the use of various educational methods with emphasis on their patients' practices may be very beneficial to their health state. It is recommended to perform the necessary modifications before utilizing this questionnaire in other countries. Satisfying results for content validity indices and reliability analysis of the developed instrument gives us confidence that it can be used as a valid and reliable tool for evaluating patients' knowledge towards beta thalassemia major. The results of this study confirm that content validity assessment can be a useful method in developing new instruments in health services research. The main limitation of current study was, due to the blood transfusion appointments and the referral of the patients to the clinic, the patients' response was challenging.

It was concluded that TMQ might be used as an instrument to measure knowledge, and practice about medications among major thalassemia patients. The reason for this conclusion is the diverse range of questions domains involved in the TMQ and the simplicity, validity, reliability and practicability of the questionnaire. Now, TMQ is available for health researchers, stakeholders, and policy makers to help them

in finding a more accurate estimation on knowledge and practice among thalassemia patients. Major thalassemia treatment has a huge health budget in Iran and measuring the knowledge and practice of these patients could lead the policy makers to invest on increasing the awareness of the patients in order to increase their adherence and better self-monitoring to decrease their disease burden in the health policies. Moreover, this questionnaire may be considered as a basis to design new tools for specific diseases in Iran.

References

1. Karimi M, Rasekhi AR. Efficiency of premarital screening of beta-thalassemia trait using MCH rather than MCV in the population of Fars Province, Iran. Haematologia. 2002;32(2):129-33.
2. Cunningham MJ, Macklin EA, Neufeld EJ, Cohen AR. Complications of beta-thalassemia major in North America. Blood. 2004;104(1):34-9.
3. Musallam KM, Cappellini MD, Taher AT. Iron overload in beta-thalassemia intermedia: an emerging concern. Curr Opin Hematol. 2013;20(3):187-92.
4. Gabutti V, Piga A. Results of long-term iron-chelating therapy. Acta Haematologica. 1996;95(1):26-36.
5. Galanello R. Deferiprone in the treatment of transfusion-dependent thalassemia: a review and perspective. Ther Clin Risk Manag. 2007;3(5):795.
6. Eleftheriou A. Thalassemia International Federation: Guidelines for the clinical management of thalassemia. 2nd Revised edition. Nicosia (CY): Thalassaemia International Federation; 2008.
7. Cappellini MD. Long-term efficacy and safety of deferasirox. Blood Rev. 2008;22:S35-S41.
8. Pakbaz Z, Fischer R, treadwell M, et al. A simple model to assess and improve adherence to iron chelation therapy with deferoxamine in patients with thalassemia. Ann N Y Acad Sci. 2005;1054(1):486-91.
9. Vichinsky E. Iron overload and iron chelation therapy in pediatric patients. US Hematology. 2009;2(1):64-7.
10. Abu Samra O, Auda W, Kamhawy H, Al-Tonbary Y. Impact of educational programme regarding chelation therapy on the quality of life for B-thalassemia major children. Hematology. 2015;20(5):297-303.
11. Motta I, Bou-Fakhredin R, Taher AT, Cappellini MD. Beta Thalassemia: New Therapeutic Options Beyond Transfusion and Iron Chelation. Drugs. 2020;80(11):1053-1063.
12. Upadhyay SC. Assessment of the knowledge of thalassemia in the thalassemia patients and the treatment received by them. The Internet Journal of Hematology. 2008;5(2):1-8.
13. Kourorian Z, Azarkeivan A, Hajibeigi B, Oshidari A, Shirkavnd A. The effect of knowledge, attitude and practice on the function of Thalassemic patients. Iranian Journal of Blood and Cancer. 2014;6(4):177-81.
14. Porteous T, Francis J, Bond C, Hannaford P. Temporal stability of beliefs about medicines: implications for optimising adherence. Patient Educ Couns. 2010;79(2):225-30.
15. Waltz CF, Bausell BR. Nursing research: design statistics and computer analysis: Davis FA; 1981.
16. Hyrkäs K, Appelqvist-Schmidlechner K, Oksa L. Validating an instrument for clinical supervision using an expert panel. Int J Nurs Stud. 2003;40(6):619-25.
17. Lawshe C. A quantitative approach to content validity. Pers Psychol. 1975;28(4):563–575.
18. Karimzaei T, Masoudi Q, Shahrakipour M, Navidiyan A, Jamalzae AA, Zoraqi Bamri A. Knowledge, attitude and practice of carrier thalassemia marriage volunteer in prevention of major thalassemia. Glob J Health Sci. 2015;7(5):364-70.
19. Poengoet BNSR, Sungkar E, Pandji TD. Quality of Life in Thalassemia Major Patients: Reliability and Validity of Indonesian Version of TranQol Questionnaire. International Journal of Integrated Health Sciences. 2017;5(2):75-9.
